# Effect of Methane Inhibitors on Ruminal Microbiota During Early Life and Its Relationship With Ruminal Metabolism and Growth in Calves

**DOI:** 10.3389/fmicb.2021.710914

**Published:** 2021-09-16

**Authors:** Omar Cristobal-Carballo, Susan A. McCoard, Adrian L. Cookson, Siva Ganesh, Katherine Lowe, Richard A. Laven, Stefan Muetzel

**Affiliations:** ^1^Ruminant Nutrition and Physiology Team, AgResearch Grasslands, Palmerston North, New Zealand; ^2^School of Veterinary Science, Massey University, Palmerston North, New Zealand; ^3^Food System Integrity, AgResearch Grasslands, Palmerston North, New Zealand; ^4^Biostatistics Team, AgResearch Grasslands, Palmerston North, New Zealand

**Keywords:** methane inhibitors, early life, rumen, metataxonomics, microbiota, imprinting, animal phenotype, fermentation profiles

## Abstract

The present study aimed to determine whether dietary supplementation with methanogen inhibitors during early life may lead to an imprint on the rumen microbial community and change the rumen function and performance of calves to 49-weeks of rearing. Twenty-four 4-day-old Friesian x Jersey cross calves were randomly assigned into a control and a treatment group. Treated calves were fed a combination of chloroform (CF) and 9,10-anthraquinone (AQ) in the solid diets during the first 12 weeks of rearing. Afterward, calves were grouped by treatments until week 14, and then managed as a single group on pasture. Solid diets and water were offered *ad libitum*. Methane measurements, and sample collections for rumen metabolite and microbial community composition were carried out at the end of weeks 2, 4, 6, 8, 10, 14, 24 and 49. Animal growth and dry matter intake (DMI) were regularly monitored over the duration of the experiment. Methane emissions decreased up to 90% whilst hydrogen emissions increased in treated compared to control calves, but only for up to 2 weeks after treatment cessation. The near complete methane inhibition did not affect calves’ DMI and growth. The acetate:propionate ratio decreased in treated compared to control calves during the first 14 weeks but was similar at weeks 24 and 49. The proportions of *Methanobrevibacter* and *Methanosphaera* decreased in treated compared to control calves during the first 14 weeks; however, at week 24 and 49 the archaea community was similar between groups. Bacterial proportions at the phylum level and the abundant bacterial genera were similar between treatment groups. In summary, methane inhibition increased hydrogen emissions, altered the methanogen community and changed the rumen metabolite profile without major effects on the bacterial community composition. This indicated that the main response of the bacterial community was not a change in composition but rather a change in metabolic pathways. Furthermore, once methane inhibition ceased the methanogen community, rumen metabolites and hydrogen emissions became similar between treatment groups, indicating that perhaps using the treatments tested in this study, it is not possible to imprint a low methane microbiota into the rumen in the solid feed of pre-weaned calves.

## Introduction

The rumen harbors a symbiotic community of microorganisms that degrade ingested plant components (Hobson and Stewart, 1997). Complex carbohydrates are hydrolyzed and fermented into short chain fatty acids (SCFA), mostly absorbed across the rumen wall and utilized as energy sources for the host ruminant (Van Soest, 1994). A by-product of acid formation in the rumen is hydrogen (H_2_) that is converted to methane (CH_4_), a potent greenhouse gas (Ellis et al., 2007). For decades, manipulations of the rumen microbiota have been attempted with the aim to improve animal performance or reduce CH_4_ emissions (Haque, 2018). In adult ruminants, microbial manipulation, e.g., dietary interventions, microbial inhibitors, plant extracts, among others, have shown either no or only short-term post-treatment effects because of the well-established rumen microbiota (Weimer et al., 2010; Weimer, 2015). In young ruminants, it has been observed that microbial establishment progresses as solid feed intake increases and the rumen develops (Fonty et al., 1987; Jami et al., 2013; Rey et al., 2014; Dill-McFarland et al., 2017). Therefore, manipulations of the rumen microbiota during early life could be a feasible mechanism to promote changes in the community structure that will persist in later life (Yáñez-Ruiz et al., 2010; Abecia et al., 2014a, b; Belanche et al., 2019; Meale et al., 2021).

Dietary and chemical interventions during early life have been shown to alter rumen microbial composition and influence CH_4_ emissions and SCFA production during and for up to 3 months after treatment cessation in small ruminants (Abecia et al., 2013, 2014b; Saro et al., 2018; Wang et al., 2019). In calves, dietary manipulations during early life have revealed that shifts in the ruminal bacterial community correlate to changes in fermentation patterns and the colonization by archaeal microorganisms (Dias et al., 2017). Such studies have also indicated that changes in the rumen microbial composition can persist to adulthood (Dill-McFarland et al., 2019). The intake of methane inhibitors, targeting the methyl-coenzyme M reductase, from birth until 3 weeks post-weaning have shown long-lasting changes for up to at least 1 year of life on the ruminal microbial ecosystem and CH_4_ emissions, without differences in live weight (LW), Average daily gain (ADG) and SCFA production between treatments (Meale et al., 2021). Overall, findings in young ruminants suggest that alterations in the early establishment of rumen microbiota may influence the microbial succession process. Additionally, early life intervention studies have reported decreased methanogenesis in the short- and long-term post-treatment, with promising mitigation results in calves maintained throughout the first year of the animals’ life. However, there is a need for more studies assessing the impact of microbial manipulation during early life on the long-term microbial establishment (bacteria and archaea), rumen function and performance in calves.

In a normal functioning rumen, H_2_ released during rumen microbial fermentation is used by methanogens to reduce carbon dioxide (CO_2_) to CH_4_ (Ungerfeld and Kohn, 2006; Janssen, 2010). Methanogenic archaea make up only 3–4% of the rumen microbial population (Yanagita et al., 2000; Ziemer et al., 2000), but they play an important role in H_2_ removal (Wolin et al., 1997; Joblin, 1999). The use of methanogen inhibitors in ruminants has shown to increase H_2_ concentrations in the rumen (Bauchop, 1967; Kung et al., 2003), change the feed fermentation pathways toward production of less acetate and more propionate and butyrate, and change the composition of the methanogen community away from the dominant *Methanobrevibacter* species (Knight et al., 2011; Martinez-Fernandez et al., 2016). However, it is not clear if alterations of the methanogen community during the first weeks of life through methane inhibitor treatment affects subsequent H_2_ emissions and succession of methanogen microbes following discontinuation of treatment.

Studies *in vitro* and *in vivo* have shown that chloroform (CF) and 9,10-anthraquinone (AQ) are potent methanogen inhibitors (Bauchop, 1967; Garcia-Lopez et al., 1996; Kung et al., 2003; Knight et al., 2011). The mechanism of action of CF on methanogens has not been confirmed. Available data indicates that CF interferes at the cobamide-dependent methyl transferase step of the methanogenesis pathway, but there could also be collateral inhibition of methyl transferases in other bacteria (Gunsalus and Wolfe, 1978; Graham and White, 2002). In contrast, AQ seems to interfere with the methyl-coenzyme M of methanogens by disrupting electron transfer during CH_4_ formation (Garcia-Lopez et al., 1996; Kung et al., 1998). The use of these methane inhibitors can have adverse effects on feed intake, digestion, rumen fermentation and LW gain when added at high concentrations (Kung et al., 2003; Martinez-Fernandez et al., 2016) with these studies examining changes in methanogen populations, ruminal fermentation and CH_4_/H_2_ production in mature ruminants. To our knowledge, there is no evidence of the effects of feeding two methanogen inhibitors (CF and AQ) during early and adult life on rumen microbial establishment, rumen function and performance in calves. The use of CF and AQ was to provide a methane inhibition effect that would last over the treatment period even if the rumen community adapts to one of the inhibitors. The objective of the present study was to determine whether feeding CF and AQ in the solid feed diet during early life may lead to an imprint on the rumen microbial community, change the fermentation pathways and alter growth performance of calves to 49 weeks of age.

## Materials and Methods

### Experimental Design

Twenty-four female dairy calves (Friesian x Jersey cross) were randomly allocated, following a simple randomization procedure, to a control and a treatment group. The treatment group had the methane inhibitors CF and AQ mixed into their starter concentrate and partial mixed ration (PMR) diets. The control diet did not contain methane inhibitors. The treatment period lasted for 12 weeks followed by a 37-week period in which both groups were fed similar diets. Animal manipulations were reviewed and approved (AE13132) by the Grasslands Animal Ethics Committee and complied with the institutional Codes of Ethical Conduct for the Use of Animals in Research, Testing and Teaching, as prescribed in the New Zealand Animal Welfare Act of 1999 and its amendments.

### Animal Management

Calves were sourced from a single commercial farm at 4 days of age and an average weight of 33 ± 3.7 kg (mean ± S.D.). On arrival to the animal facility, calves were weighed and assigned to one of the experimental groups. Calves were housed in individual pens (1.5 m × 3 m) bedded with wood shavings. Experimental groups were housed in separate temperature-controlled rooms to avoid cross contamination of ruminal microbes between treatments. At week 10, after weaning off milk, calves were moved from individual to a single group pen within each treatment group. At week 14, calves from both groups were transferred outdoors and managed as one mob on pasture. LW was determined weekly during the first 10 weeks of life, fortnightly until week 24 of age, and monthly thereafter. ADG was calculated at the end of the experiment.

### Feeding Management and Diet Composition

All calves were fed 4 L/d (2 meals of 2 L per day) of colostrum during the first 4 days of life prior to enrollment into the trial. From day 5 onward, calves were fed 4 L of reconstituted milk replacer (MR; 125 g/kg dry matter: 20.6% fat, 22.7% crude protein (CP) and 49.5% lactose; Milligans, Oamaru, New Zealand) split into two equal feeds of 2 L fed at 0800 and 1600 h using individual feeders. At week 4, calves were transitioned to once per day milk feeding, where 4 L was offered in the morning only. At week 10, calves were fully weaned from MR over a 10-day period reducing individual milk intake by 10% per day. Starter concentrate (Denver Stock Feeds, Palmerston North, New Zealand) was offered *ad libitum* from day 8. From week 4, calves were offered *ad libitum* access to starter concentrate plus a PMR. Calves were weaned off starter concentrate from weeks 12 to 14. At week 14, calves were moved outdoors and managed as a single mob on a ryegrass and red clover mixed sward, with continue free access to the PMR. Calves were weaned off PMR diet from week 15 to 17, reducing intake by 10% per day. Fresh water was available *ad libitum* throughout the study.

The ingredients used in the starter concentrate and PMR diets are given in Table 1. The chemical composition of the milk replacer, starter concentrate, PMR diet and pasture diets (Table 2) was determined by wet chemistry at the Nutrition Lab at Massey University (Palmerston North, New Zealand). Compositional analyses were carried out according to the methods of the Association of Official Analytical Chemists (AOAC, 1990, 2010, 2012). When animals were grazed, pasture samples were taken during methane measurements and scanned by near-infrared reflectance spectroscopy (NIRS; FeedTECH, AgResearch Ltd., Palmerston North, New Zealand) for ash, ether extract, CP, neutral detergent fiber (NDF) and water soluble carbohydrates (WSC) contents (Corson et al., 1999). Dry matter intake (DMI) of starter concentrate and PMR diets were measured on a daily basis only during the individual housing period. Additionally, DMI of starter concentrate, PMR and pasture were also determined when animals were brought into the respiration chambers to measure methane emission. The feed intake was calculated as the difference between feed offered and refused by the animal. Dry matter content was determined by drying the feed offer and refusal for 48 h at 105°C.

**TABLE 1:** Ingredients of the starter concentrate and the partial mixed ration (PMR) diet.

Starter concentrate	g/kg	PMR diet	g/kg
Maize	108	Chopped hay	500
Barley	432	Barley	290
Peas	173	Soya	100
Soya	205	Molasses	100
Molasses	54	Di-calcium-phosphate	5.5
Sodium bicarbonate	20	Salt	3.0
Salt	5.0	Mineral/vitamin mix	1.5
Calf pre-mix	1.0		
Bovatec	0.6		
Rumasweet palatant	0.2		

**TABLE 2:** Chemical composition (g/kg) of the milk replacer, starter concentrate, partial mixed ration (PMR) diet and pastures.

Diet	Milk replacer^1^	Starter concentrate	PMR diet	Pasture Wk24^a^	Pasture Wk49^a^
Dry matter^2^	97.0	88.7	82.8	17.6	20.2
Crude protein^3^	22.7	20.8	13.3	21.8	18.9
WSC^4^	49.5*	56.1	5.1	11.6	8.7
NDF^5^	0.0	15.5	43.8	43.3	49.3
Ether extract^6^	20.6	1.7	1.1	3.5	2.1
Ash^7^	6.2	5.9	5.6	9.5	9.2

*^*a*^Chemical composition of pastures was scanned using the scanned by near-infrared reflectance spectroscopy (NIRS; Corson et al., 1999).*

*^1^Manufacturers data.*

*^2^Method 945.15; AOAC, 2010.*

*^3^Method 992.15; AOAC, 2010.*

*^4^Water soluble carbohydrates (WSC); (Paul and Southgate, 1978).*

*^5^Neutral detergent fiber (NDF); method 7.074; AOAC, 1990.*

*^6^Method 954.02; AOAC, 1990.*

*^7^Method 942.05; AOAC, 2012.*

*Lactose.

### Methane Inhibitors

The inhibitors used in this study were 9,10-AQ (A90004, Sigma-Aldrich, St Louis, MO, United States) and CF (C2432, Sigma-Aldrich, St Louis, MO, United States). The CF was complexed with β-cyclodextrin (β-CD; Trappsol^®^, TBCDF-F, Cyclodextrin Technologies Development Ltd., Gainesville, FL, United States) to stop evaporation from the feed and to render it odorless. 50 ml of CF were added to β-CD dissolved at 10% (w/v) in 10 L of water. The mix was kept at 4°C and shaken every hour during an 8 h period, after which it settled over night. The supernatant liquid was decanted and the sediment filtered using Whatman no. 54 filter paper, dried in the fridge at 4°C and transferred to a jar. Concentrations of 9,10-AQ and CF fed to the calves in the starter concentrate and PMR diets were 500 and 50 mg/kg of feed, respectively. The choice and dose of the CF/AQ mix was based on a dose response test run *in vitro* (Muetzel, personal communication). Both AQ and CF were pre-mixed into approximately 5 kg of starter concentrate using a food processor and then mixed into the total batch amount using a concrete mixer. The same process was used for the PMR diet, where the inhibitors were first mixed into 5 kg of soybean meal, then into the total soybean meal using a concrete mixer and, finally, the rest of the ingredients were incorporated in a large mixer. The final mixes were prepared twice a week and stored at 4°C until used.

### Measurements of Gas Emissions and Dry Matter Intake

Emissions of CH_4_ and H_2_ were measured at weeks 2, 4, 6, 8, 10, 14, 24 and 49. Measurements were carried out in open circuit respiratory chambers (Pinares-Patiño et al., 2012) for 24 h. The air flow through the chambers was adjusted to 600 L/min to account for the low CH_4_ emissions of a young animal. For the last two measurements, the airflow was increased to 1,000 and 1,500 L/min, respectively. Calves entered the chambers in the morning (0800 h), when solid diets, i.e., the starter concentrate and/or PMR diets (week 2–14), or fresh grass (week 24 and 29), were offered. During the milk feeding period, MR was offered before entering to the chambers in the morning (0800 h) and on weeks 2 and 4 also before the afternoon feed allocation (1600 h). For the last two measurements, the same type of pasture that the animals were consuming in their allocated paddocks was cut daily (Aorangi Farm, AgResearch, New Zealand) and transported to the Animal Facility at Grasslands. For measurements at week 24 and 49, calves were moved to indoor yards and adapted to eat fresh cut pasture in pens for five to 7 days prior to entering to the chambers. Animals in the chambers were offered solid diets *ad libitum* and refusals were collected to determine DMI from the difference between feed allowance and refusals.

### Rumen Fluid Sampling

Rumen samples were taken via stomach tubing after removing the calves from the respiration chambers (weeks 2, 4, 6, 8, 10, 14, 24 and 49). Each sample was subsampled for SCFA analysis (1.8 ml) and DNA extraction (0.9 ml). Samples for SCFA analysis were centrifuged (20,000 × *g*, 10 min, 4°C) and an aliquot of 0.9 ml of the supernatant was collected into 0.1 ml of internal standard (19.8 mM ethylbutyrate in 20% v/v phosphoric acid) and stored at −20°C until analysis. Rumen samples for subsequent DNA extraction and microbial community analysis were snap-frozen and stored at −20°C.

### Short Chain Fatty Acid Analysis

Samples for SCFA were thawed and centrifuged (20,000 × *g*, 10 min, 4°C) and 0.8 ml of the supernatant was collected into a crimp cap glass vial. Gas chromatography was used to analyze SCFA composition (Attwood et al., 1998) in a HP 6,890 gas chromatograph equipped with a flame ionization detector using a Zebron ZB-FFAP 30.0 m × 0.53 mm I.D × 1 μm film column (Tavendale et al., 2005).

### DNA Extractions, Amplification of Target Genes and Amplicon Pooling

Nucleic acids were extracted from 200 μl of the rumen fluid using the phenol-CF, bead beating, with filtration kit for purification II (PCQI) method (Henderson et al., 2013; Kittelmann et al., 2013). A total of 120 ng of DNA contained in 6 μl of water were divided into 3 aliquots of 20 μl each (Kittelmann et al., 2013). DNA extracts were quantified on a FlexStation 3 (Molecular Devices, LLC. San Jose, CA, United States) and run on a 1% agarose gel with a lambda-*Hin*dIII marker to determine sizing and integrity. DNA primers are presented in Supplementary Table 1. PCR amplicon reactions (30 and 35 cycles for bacteria and archaea, respectively), targeting the region of the 16S rRNA genes in the microbial groups bacteria and archaea, were prepared as described by Kittelmann et al. (2013), with the following modifications. Triplicate PCR products were pooled, and the correct sizes of PCR amplicons and the absence of signal from negative controls were verified by agarose gel electrophoresis and quantified by fluorescence using the Quant-iT dsDNA BR assay kit (Invitrogen, Carlsbad, CA, United States). For each amplicon, 150 ng from the same target gene and region (i.e., all bacteria and archaea amplicons) were pooled, concentrated and quantified (Quant-iT dsDNA HS assay kit; Invitrogen, Carlsbad, CA, United States). Each pool was then purified using the NucleoMag NGS kit (Macherey-Nagel, Dueren, Germany), with a final purification of the amplicons performed with the QIAquick PCR Purification kit (Qiagen, Valencia, CA, United States). The resulting DNA concentration was quantified using Quant-iT dsDNA HS assay kit (Invitrogen, Carlsbad, CA, United States). Both pools were then diluted to 6.0 × 10^9^ copies per μl and combined at a bacteria to archaea ratio of 5:1 (Kittelmann et al., 2013).

Before sequencing, pooled libraries were checked for quality control (QC) with the Labchip GX Touch HT Instrument (PerkinElmer, Waltham, MA, United States) using the DNA High Sensitivity assay. Amplicons were sequenced at the Massey Genome Service/New Zealand Genomics Limited using Illumina MiSeq system (Massey University, Palmerston North, New Zealand). The pooled library was run on one Illumina MiSeq; 2 × 250 base PE run version 2 chemistry (Reagent Kit v2, 500 cycles; Invitrogen, Carlsbad, CA, United States). An Illumina prepared PhiX control library for the run was loaded onto the Illumina MiSeq run at 20% volume. Sequence reads were provided in fastq format. Raw sequence reads were deposited in the European Nucleotide Archive under the accession number PRJEB37781.

### Phylogenetic Analysis

Sequencing reads were quality-filtered using the DynamicTrim function of SolexaQA (Cox et al., 2010). Reads were then processed and analyzed using QIIME version 1.8 (Caporaso et al., 2010). Sequencing reads were grouped, using the UCLUST algorithm, into operational taxonomic units (OTUs) sharing similarities over 97% for bacteria and 99% for archaea (Edgar, 2010). Sequences were then assigned to phylogenetic groups using the BLAST (version 2.4.0) algorithm (Altschul et al., 2012). Bacterial 16S rRNA genes were assigned using SILVA 123 (Henderson et al., 2019) and archaea 16S rRNA genes using RIM-DB (Seedorf et al., 2014). OTU-tables generated by QIIME were used for downstream statistical analysis.

### Statistical Analysis

The effects of including CF and AQ (methane inhibitors) in the solid feed diet of calves from 1 to 12 weeks of age on DMI, ADG and LW, rumen function (SCFA and enteric emissions) and microbial community composition (bacteria and archaea) were evaluated during and after treatment.

The effects of treatment on rumen function, and DMI, ADG and LW were analyzed by fitting a linear mixed effect (LME) model via the restricted maximum likelihood (REML) framework as implemented in the *NLME* package in R (Pinheiro et al., 2015; R Core Team, 2016). DMI, LW, ADG, CH_4_, and H_2_ emissions, total concentrations and individual proportions of SCFA were fitted in an LME model and included treatment and time as fixed effects and animal as random effect. LW was adjusted to initial LW at trial entry. The equation used is as follows:


$${\text{y}}_{\text{ijk}}=\mu +\beta_{\text{k}}+\alpha_{\text{i}}+\gamma_{\text{j}}+{\left(\alpha \gamma \right)}_{\text{ij}}+{\text{u}}_{\text{ik}}+{\text{e}}_{\text{ijk}}$$

where y_ijk_ is response at time j for the kth animal in the ith treatment, μ is the general mean, β_k_ is the covariate for the animals within treatments (only used for analysis of LW), α_i_ is the effect of the ith treatment, γ_j_ is the effect of the jth time, (αγ)_ij_ is the interaction between treatment and time, u_ik_ is the normally distributed random experimental error for the experimental units (the animals within treatments) with constant variance σ_u_^2^, and e_ijk_ is the normally distributed random experimental error on repeated measures with variance σ_e_^2^. The resulting models were analyzed by repeated-measures ANOVA and Tukey’s post-test to determine the longitudinal effects of the intake of methanogen inhibitors during early life. Predicted means from the model, together with estimates of the standard error of the mean (SEM), permutation *F*-test of the model (1,000 permutations), and pairwise comparisons (Tukey test) were obtained using the *PREDICTMEANS* package in R (Luo et al., 2014). Significance was declared when *P* ≤ 0.050 (Ganesh and Cave, 2018).

The OTU-tables of the rumen microbial community generated by QIIME were analyzed to determine changes in the alpha and beta diversity of the bacteria and archaea communities produced by treatment interventions. The alpha diversity of the rumen bacterial and archaeal microbiota were analyzed using the Shannon index in the *VEGAN* package of R (Oksanen et al., 2017). The effects of methanogen inhibitors on the Shannon index of the microbial (bacterial and archaeal) community were analyzed using an LME model and repeated measurements ANOVA as described for rumen function, and DMI and ADG data. Predicted means from the models, together with estimates of the SEM were obtained, and pairwise comparisons were done using Tukey’s test. The beta diversity of the entire community and abundant community of bacteria and archaea (abundant microbes are described below) were analyzed using the partial least squares discriminant analysis (PLSDA) in the *MixOmics* package of R (Lê Cao et al., 2016).

Univariate analyses were done to identify the effects of methane inhibitors in the abundant bacteria and archaea taxa. Abundant bacterial phyla, bacterial genera and archaea species were defined as organisms with an average proportion ≥0.5, ≥0.5 and ≥1.0%, respectively, across the complete dataset. The effect of treatment on the abundant bacterial and archaeal communities was analyzed using an LME model and repeated measurements ANOVA as indicated for rumen function and animal DMI and ADG. After checking for normality, the data of the most abundant bacterial and archaeal taxa were natural logarithm transformed. Predicted means from the model, together with the 95% confidence interval (CI) of the geometric mean, permutation *F*-test of the model, and pairwise comparisons were obtained, and back transformed using the *PREDICTMEANS* package of R. Pairwise *P*-values were calculated using the Benjamini–Hochberg test (BH; Benjamini and Hochberg, 1995). Transformed mean and 95% CI were reported as indicated by Bland and Altman (1996). Significance was declared when *P* ≤ 0.050.

## Results

### Animal Performance

**FIGURE 1:**
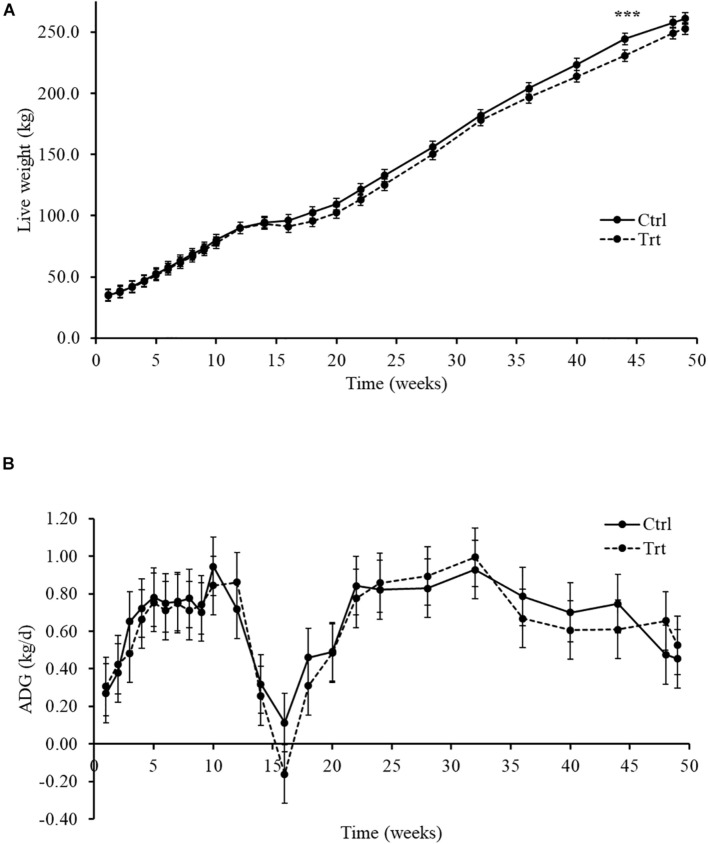
Growth performance from week 1 to 49 in control group (Ctrl) and treated (Trt) calves. Growth performance was measure as: **(A)** Live weight (kg) and **(B)** average daily gain (ADG; kg/d). Calves during the rearing time were fed as follows: milk twice a day and *ad libitum* control and treatment concentrates at weeks 2 and 4; milk once a day and *ad libitum* control and treatment concentrates and partial mixed ration (PMR) diet at weeks 6, 8, and 10; concentrates step-down weaned and PMR diets fed *ad libitum* until week 14; and grazing a mixed sward of ryegrass/clover as one mob at weeks 24 and 49. Predicted means and their least square of the difference (LSD) are presented. Significance of pairwise comparisons (Tukey post-hoc analysis) between treatments are indicated by asterisks as: ***P < 0.001.

The effect of methane inhibitors inclusion in the solid feed diet on DMI for treated and control calves is shown in Table 3. DMI of starter concentrate, PMR, pasture and total DMI of all solid feed diets increased over time (*P* < 0.001) in both treatment groups. Total mixed ratio intake was 11% lower (*P* = 0.022) in treated compared to control calves. Concentrate intake had a treatment by time interaction effect tendency (*P* > 0.090), but the *post hoc* (Tukey test) analysis did not show differences (*P* ≥ 0.468) between treated and control calves. No other treatment by time effects were observed for PMR, pasture and total DMI of all solid feed diets. LW and ADG of treated and control calves is presented in Figure 1. There was a treatment by time interaction (*P* < 0.001) for overall LW gain throughout the study, nevertheless this was primarily influenced by a 5.6% difference in LW between treated and control calves at week 44 (231 kg vs. 245 kg, *P* < 0.001; Figure 1A). However, there was no evidence (*P* = 0.087) of an effect of treatment on ADG (Trt = 0.613 kg/d [C.I. = 0.590 – 0.637] vs. Ctrl = 0.642 kg/d [C.I. = 0.619 – 0.666]). ADG declined from week 14 to 18 following removal of the starter concentrate with recovery of growth rates observed by week 20 (Figure 1B).

**TABLE 3:** Effect of methane inhibitors^1^ on dry matter intake (DMI).

		Rearing period (weeks)														P-val		
	Treatment	1	2	3	4*	5	6	7	8	9	10	14	24	49	SED	P-Tx	P-Tm	P-Int
Concentrate (kg)	Ctrl	0.11	0.24	0.37	0.54	0.67	0.68	0.75	0.74	0.81	0.82	–	–	–	0.046	0.117	<0.001	0.090
	Trt	0.09	0.21	0.34	0.46	0.53	0.57	0.61	0.66	0.75	0.83	–	–	–				
PMR (kg)	Ctrl	–	–	–	0.17	0.21	0.37	0.54	0.73	1.04	1.21	2.38	–	–	0.062	0.022	<0.001	0.150
	Trt	–	–	–	0.15	0.21	0.30	0.43	0.57	0.83	1.09	2.33	–	–				
Pasture (kg)	Ctrl	–	–	–	–	–	–	-	–	–	–	–	3.05	6.84	0.466	0.524	<0.001	0.360
	Trt	–	–	–	–	–	–	-	–	–	–	–	2.87	7.28				
Total DMI (kg)	Ctrl	0.11	0.24	0.37	0.65	0.88	1.05	1.29	1.47	1.85	2.03	2.38	3.05	6.84	0.193	0.396	<0.001	0.572
	Trt	0.09	0.21	0.34	0.59	0.75	0.87	1.04	1.23	1.58	1.93	2.33	2.87	7.28				

*DMI was calculated for starter concentrate, partial mixed rations (PMR), pasture and total DMI in dairy calves. Results^2^ are the means and standard error of the differences (SED), P-value for treatment (P-Tx), time (P-Tm) and interaction (P-Int). Significance of pairwise comparisons (Tukey post hoc analysis) between treatments are shown in bold at each sampling time.*

*^1^The treatment involved dosing a mix of 9,10-anthraquinone (AQ) and chloroform (CF) at 500 and 50 mg/kg of feed, respectively, from arrival until week 12 of the rearing period, after which both groups were on the same control diet.*

*^2^Repeated measurements were used to analyze the long-term effects of the methane inhibitors in the rumen function of calves.*

*Calves were introduced to PMR diet during week 4, where 4 control and 3 treated calves did not consumed PMR; therefore, the mean of the total DMI does not correspond to the sum of the means of concentrate and PMR.

### Rumen Fermentation

The intake of methane inhibitors on the rumen function is shown in Table 4. Treatment by time interaction effects (*P* ≤ 0.001) were evident during the first 14 weeks of rearing for yield and production of CH_4_ and H_2_, and the percentage of acetate, propionate, caproate, valerate and isovalerate. Treated calves had average CH_4_ yield decreases (*P* < 0.001) of 7.9 ± 1.90-fold (mean ± SEM), and H_2_ yield (*P* < 0.001) increases of 88.7 ± 48.78-fold compared to control calves, but similar values for CH_4_ yield and H_2_ yield in both groups at 24 and 49 weeks. The average proportion of acetate decreased 1.2 ± 0.02-fold (*P* < 0.001) and those of propionate increased 1.3 ± 0.05-fold (*P* = 0.001) in treated compared to control calves, but the proportions of butyrate were similar (*P* = 0.246) in both groups. When CH_4_ was inhibited, treated calves had valerate 2.1 ± 0.17-fold greater (*P* < 0.001), caproate 2.8 ± 0.08-fold greater (*P* < 0.001) and isovalerate (*P* < 0.001) 2.4 ± 0.19-fold greater average proportions than control calves. No interaction effects were observed for isobutyrate (*P* = 0.462) or total SCFA concentrations (*P* = 0.723). Similar SCFA profiles were observed in both groups at weeks 24 and 49.

**TABLE 4:** Effect of methane inhibitors^1^ on rumen function^2^.

		Rearing period (weeks)									*P*-value		
	Treatment	2	4	6	8	10	14	24	49	SED	P-Tx	P-Tm	P-Int
Methane production (g/d)	Ctrl	1.53	**7.44**	**19.04**	**25.80**	**44.04**	**50.81**	63.80	145.40	3.037	<0.001	<0.001	<0.001
	Trt	0.69	**1.04**	**1.50**	**3.81**	**2.70**	**12.28**	58.76	143.13				
Methane yield (g/kg DMI)	Ctrl	5.16	**15.72**	**25.80**	**23.99**	**20.20**	**21.08**	22.33	21.86	2.161	<0.001	<0.001	<0.001
	Trt	4.58	**4.38**	**2.27**	**3.85**	**1.41**	**5.39**	21.08	20.26				
Hydrogen production (g/d)	Ctrl	0.17	0.14	**0.42**	**0.07**	**0.02**	**0.04**	0.08	0.29	0.388	<0.001	<0.001	<0.001
	Trt	0.35	0.82	**2.48**	**3.29**	**5.93**	**4.35**	0.09	0.44				
Hydrogen yield (g/kg DMI)	Ctrl	0.98	**0.38**	**0.89**	**0.07**	**0.01**	**0.02**	0.03	0.07	0.437	<0.001	<0.001	<0.001
	Trt	1.68	**2.64**	**3.41**	**2.93**	**2.97**	**1.88**	0.02	0.06				
SCFA (mM)	Ctrl	81.60	77.36	76.26	80.46	85.48	80.17	75.47	74.39	7.190	0.313	0.667	0.723
	Trt	72.57	84.39	78.00	73.13	79.50	71.69	74.47	72.57				
Acetate (%)	Ctrl	**53.67**	**54.50**	**62.92**	**61.86**	**62.66**	**67.21**	67.56	69.61	2.131	<0.001	<0.001	<0.001
	Trt	**48.71**	**44.82**	**47.77**	**47.71**	**49.11**	**52.46**	67.73	69.22				
Propionate (%)	Ctrl	**26.01**	**26.53**	**22.23**	**23.22**	**22.36**	**17.73**	18.63	17.68	2.115	<0.001	<0.001	0.001
	Trt	**32.90**	**35.45**	**27.78**	**28.76**	**33.73**	**25.54**	18.47	18.15				
Butyrate (%)	Ctrl	15.18	13.46	10.09	**10.06**	10.67	10.55	9.80	8.42	2.198	0.154	0.011	0.246
	Trt	12.83	12.82	14.05	**14.89**	11.12	13.82	9.77	8.48				
Caproate (%)	Ctrl	1.17	0.64	**0.68**	**0.67**	0.78	0.58	0.38	0.25	0.267	0.027	<0.001	<0.001
	Trt	0.93	0.76	**1.81**	**1.94**	1.14	0.74	0.33	0.25				
Valerate (%)	Ctrl	2.72	**2.55**	**1.89**	**1.62**	**1.69**	**1.47**	1.13	1.06	0.366	<0.001	<0.001	<0.001
	Trt	3.05	**3.87**	**4.80**	**3.94**	**3.48**	**2.79**	1.13	1.03				
Isobutyrate (%)	Ctrl	0.64	1.06	0.97	1.11	0.75	1.07	1.09	1.36	0.120	0.914	<0.001	0.462
	Trt	0.71	0.92	1.13	1.20	0.61	0.95	1.13	1.33				
Isovalerate (%)	Ctrl	0.62	1.27	**1.23**	1.46	1.09	**1.39**	1.41	1.61	0.432	0.075	<0.001	<0.001
	Trt	0.87	1.36	**2.62**	1.56	0.81	**3.69**	1.45	1.54				

*Results^3^ are the means and standard error of the differences (SED), *P*-value for treatment (P-Tx), time (P-Tm) and interaction (P-Int). Significance of pairwise comparisons (Tukey *post hoc* analysis) between treatments are shown in bold at each sampling time.*

*^1^The treatment involved dosing a mix of 9,10-anthraquinone (AQ) and chloroform (CF) at 500 and 50 mg/kg of feed, respectively, from arrival until week 12 of the rearing period, after which both groups were on the same control diet.*

*^2^Rumen Function was defined as: methane and hydrogen production (g/d) and yield (g/kg DMI), and total concentrations (mM) and individual proportions (%) of short chain fatty acids (SCFA) in dairy calves.*

*^3^Repeated measurements were used to analyze the long-term effects of the methane inhibitors in the rumen function of calves.*

### Rumen Microbiota

A total of 9,527,448 reads were obtained from 190 rumen samples, using the Illumina MiSeq platform, with an average of 42,445 bacteria and 7,699 archaea sequences per sample. The number of OTUs was 1,500 and 40 for bacteria and archaea, respectively. A total of 245 bacteria and 17 archaea taxa were analyzed after using a minimal sample cut-off of 200 reads.

**FIGURE 2:**
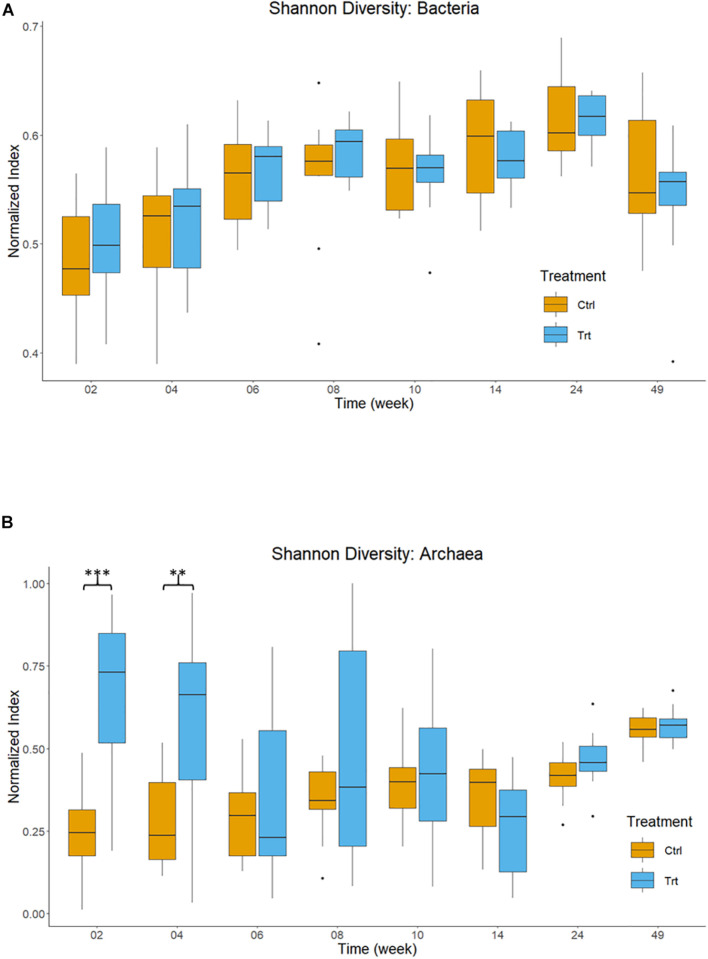
Shannon diversity indices of the rumen microbiota in control (orange) and treatment (blue) calves across different ages. Calves during the rearing time were fed as follows: milk twice a day and *ad libitum* control and treatment concentrates at weeks 2 and 4; milk once a day and *ad libitum* control and treatment concentrates and partial mixed diet (PMR) diet at weeks 6, 8, and 10; concentrates step-down weaned and PMR diets fed *ad libitum* until week 14; and grazing a mixed sward of ryegrass/clover as one mob at weeks 24 and 49. Sampling times were from 2 to 49 weeks. Shannon index for **(A)** bacteria and **(B)** archaea. Boxplots represent the 25th and 75th percentiles, lines within boxes are the medians, the whiskers extend to the most extreme data points, and dots represent the outliers. Significance of pairwise comparisons (Tukey *post hoc* analysis) between treatments are indicated by asterisks as: **P < 0.010; ***P < 0.001.

Figure 2 shows the normalized Shannon index for bacteria and archaea of treated and control calves across sampling periods. The bacterial Shannon index (Figure 2A) increased (*P* < 0.001) over time, but it was similar for the effects of treatment (*P* = 0.713) and treatment by time interaction (*P* = 0.907). The archaeal Shannon index (Figure 2B) showed an effect of treatment by time interaction effect (*P* < 0.001), where treated calves had a greater diversity at weeks 2 (*P* < 0.001) and 4 (*P* = 0.002) compared to control calves. In treated calves (0.48 ± 0.024), the archaea community showed a 24.5% greater (*P* = 0.002) Shannon index than in control calves (0.36 ± 0.024). Time effects (*P* < 0.001) were evident in calves at weeks 6 (0.33 ± 0.038) and 14 (0.30 ± 0.037) and had a lower (*P* < 0.001) archaeal diversity than calves at week 49 (0.56 ± 0.038).

**FIGURE 3:**
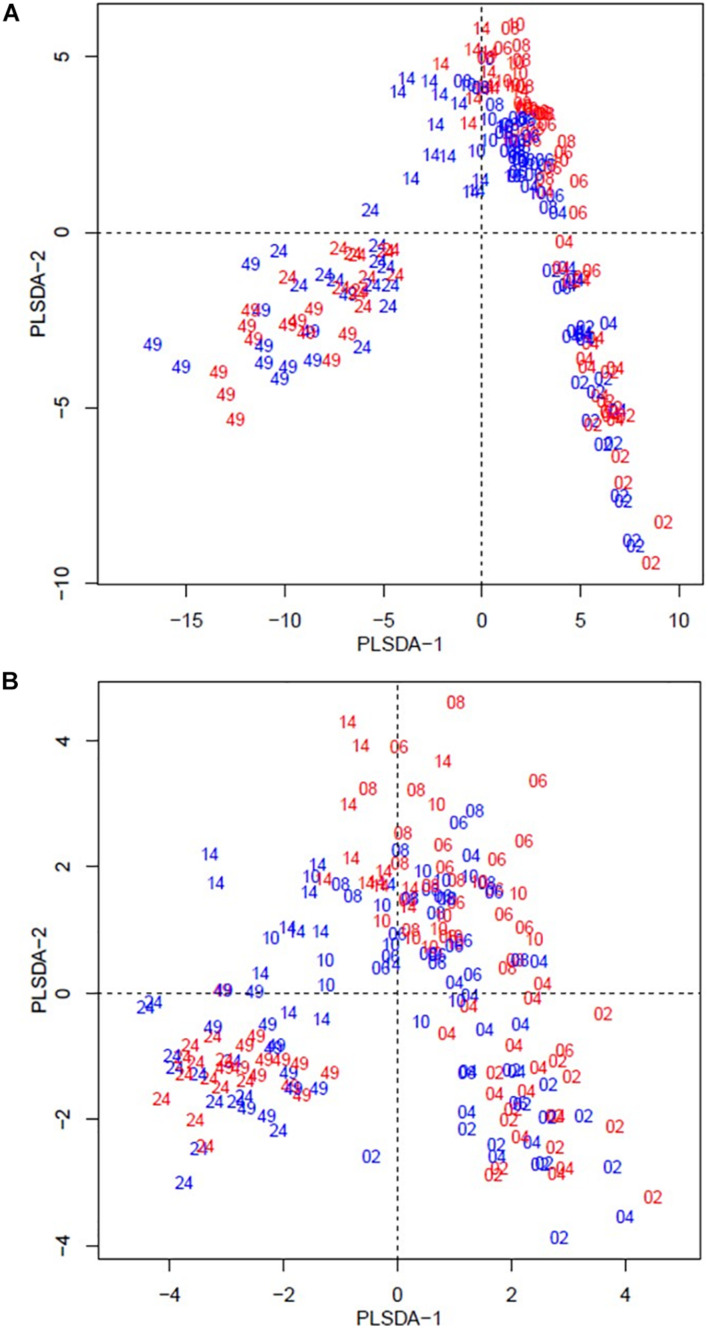
Partial least square discriminant analysis (PLSDA) of the bacteria community at the genus level in treatment (red) and control (blue) dietary treatment across sampling periods. Calves during the rearing time were fed as follows: milk twice a day and *ad libitum* control and treatment concentrates at weeks 2 and 4; milk once a day and *ad libitum* control and treatment concentrates and partial mixed diet (PMR) diet at weeks 6, 8, and 10; concentrates step-down weaned and PMR diets fed *ad libitum* until week 14; and grazing a mixed sward of ryegrass/clover as one mob at weeks 24 and 49. Sampling times were from 2 to 49 weeks. **(A)** PLSDA of the bacteria community – 245 bacterial genera. **(B)** PLSDA of the abundant ( = 0.5 %) bacteria – 41 abundant bacterial genera. Calves from the treated and control groups are represented in red and blue, respectively. The numbers correspond to the sampling time in weeks.

**FIGURE 4:**
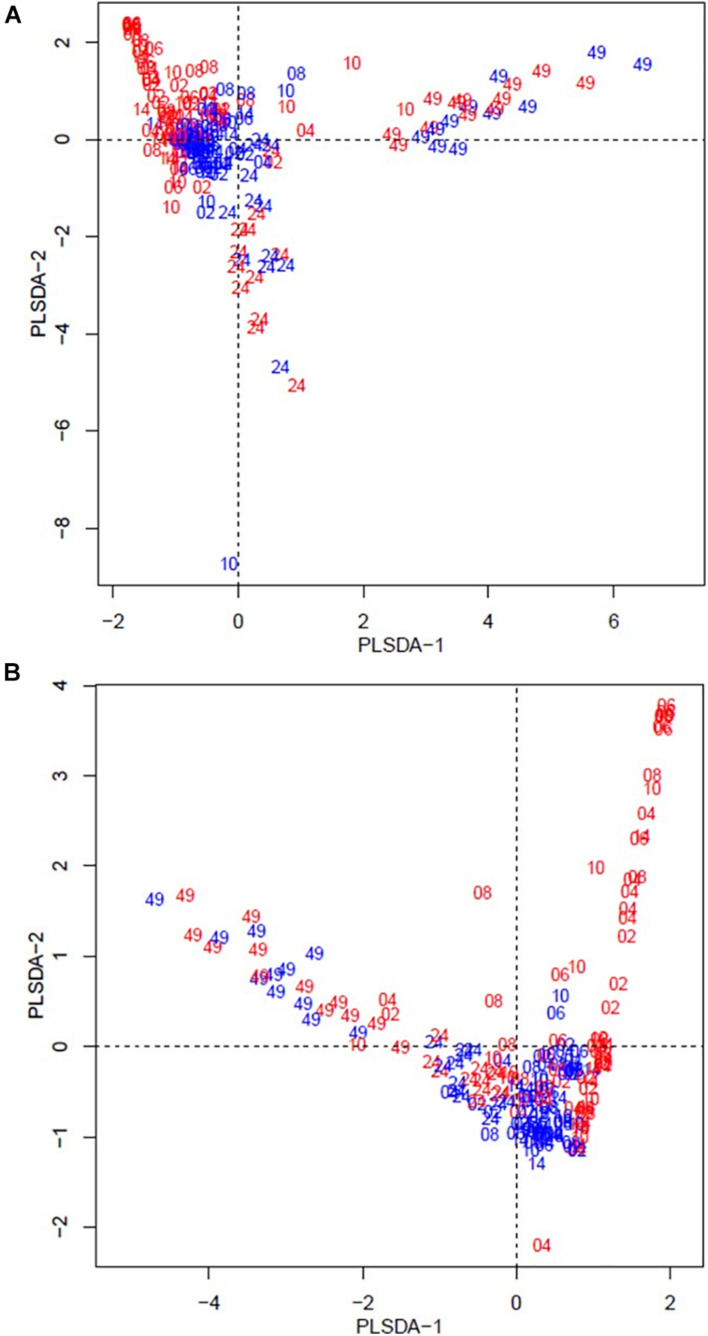
Partial least square discriminant analysis (PLSDA) of the archaea community at the species level for treated (red) and control (blue) calves across sampling times. Calves during the rearing time were fed as follows: milk twice a day and *ad libitum* control and treatment concentrates at weeks 2 and 4; milk once a day and *ad libitum* control and treatment concentrates and partial mixed diet (PMR) diet at weeks 6, 8, and 10; concentrates step-down weaned and PMR diets fed *ad libitum* until week 14; and grazing a mixed sward of ryegrass/clover as one mob at weeks 24 and 49. Sampling times were from 2 to 49 weeks. **(A)** PLSDA of the archaea community – 17 archaeal species. **(B)** PLSDA of the abundant ( = 0.5 %) archaea community – 7 archaeal species. Calves from the treated and control groups are represented in red and blue, respectively. The numbers correspond to the sampling time in weeks.

The beta diversity of the bacteria dataset is shown in Figure 3. The PLSDA of the whole dataset (245 bacterial genera) in Figure 3A shows no clustering for the treatments, but a continuum across the two dimensions according to animal age or diet. Figure 3B shows the analysis for the abundant bacteria that occurred on average at a level higher than 0.5%, where there was no clustering treatments and less clear continuum of the timeline was observed. The beta diversity of the archaeal community is shown in Figure 4. The whole archaea community and the abundant subset in treated calves clustered apart from control calves during the first 14 weeks (Figures 4A,B). While at week 24 and 49, both groups clustered together within two defined clusters in the whole archaea community (17 archaea species; Figure 4A), but in the abundant archaea (7 archaea species) only the community at week 49 of both treatments clustered separately (Figure 4B).

### Bacterial Community Composition

The bacterial community, from the selected cut offs of OTUs, showed 18 different phyla. These phyla corresponded to 32 classes, 46 orders, 81 families and 244 genera (Supplementary Tables 2–6). Phyla with a relative abundance ≥0.5% across samples showed that *Firmicutes* (43.1 ± 6.89%; mean ± S.D.) and *Bacteroidetes* (43.1 ± 7.22%) were the most abundant phyla. Whilst *Proteobacteria*, *Spirochaetae*, *Tenericutes*, *Fibrobacteres*, *Actinobacteria*, and *Cyanobacteria* together represented only 12.8 ± 4.71% of the bacteria phyla in the rumen. The effect of methane inhibitors on the abundant bacterial phyla at different sampling times is shown in Table 5. A treatment by time interaction effect was observed in *Fibrobacteres* (*P* = 0.048) and *Cyanobacteria* (*P* = 0.003). Pairwise analysis (*P* = 0.137) did not show differences for *Fibrobacteres* at any of the sampling times between treatments. The relative abundance of *Cyanobacteria* decreased in treated calves at weeks 10 and 14 (5.6- and 10.7-fold change, respectively; *P* < 0.01) with respect to control calves. Time effects were observed (*P* < 0.001) in the abundant bacterial phyla composition, for all except the *Actinobacteria* (*P* = 0.125). The proportions of *Bacteroidetes* and *Firmicutes* were constant until week 49, when *Bacteroidetes* dominated the ruminal community. Other changes over time were the general decline in *Proteobacteria* and at the same time an increase in *Spirochetes* until weaning (week 10) that decreased thereafter.

**TABLE 5:** Effect of methane inhibitors^1^ on the relative abundance of bacterial phyla (%)^2^ at different sampling times^3^.

		Time (week)									*P*-value		
Taxa-Phylum	Treatment	2	4	6	8	10	14	24	49	SED	P-Tx	P-Tm	P-Int
Firmicutes	Ctrl	44.03	37.35	41.97	36.52	46.37	48.82	53.30	30.99	5.066	0.301	<0.001	0.100
	Trt	36.61	29.24	45.48	47.67	42.04	44.23	50.59	30.10				
Bacteroidetes	Ctrl	36.17	41.46	38.54	39.92	36.51	38.57	40.22	58.75	5.133	0.264	<0.001	0.540
	Trt	37.00	46.13	37.59	35.73	43.25	42.96	41.73	59.61				
Proteobacteria	Ctrl	2.53	4.71	1.10	5.11	4.59	2.85	0.68	2.30	0.417	0.482	<0.001	0.052
	Trt	8.09	9.57	3.36	2.27	2.59	2.31	0.56	2.09				
Spirochaetae	Ctrl	0.15	0.51	2.90	4.21	1.70	0.68	0.77	0.89	0.172	0.535	<0.001	0.264
	Trt	0.35	0.77	1.80	2.14	2.63	1.28	0.91	0.93				
Tenericutes	Ctrl	0.12	0.24	0.34	0.37	0.62	1.44	1.42	1.34	0.096	0.625	<0.001	0.771
	Trt	0.06	0.23	0.34	0.60	0.81	1.84	1.49	1.38				
Fibrobacteres	Ctrl	0.01	0.04	0.22	0.27	0.76	0.65	0.39	1.03	0.053	0.751	<0.001	0.048
	Trt	0.03	0.09	0.04	0.17	0.86	0.21	0.58	1.63				
Actinobacteria	Ctrl	0.78	0.62	0.42	0.49	0.49	0.59	0.48	0.50	0.065	0.574	0.125	0.126
	Trt	0.33	0.76	0.24	0.60	0.90	0.44	0.55	0.36				
Cyanobacteria*	Ctrl	0.01	0.01	0.06	0.11	**0.28**	**0.75**	0.36	0.91	0.030	0.001	<0.001	0.003
	Trt	0.01	0.01	0.02	0.04	**0.05**	**0.07**	0.27	0.95				

*Calves were arranged in a control (Ctrl) and treatment (Trt) group. Results^4^ are natural log back transformed means and standard error of the differences (SED), *P*-value for treatment (P-Tx), time (P-Tm) and interaction (P-Int). Significance of pairwise comparisons (Benjamini–Hochberg test) between treatments are shown in bold at each sampling time.*

*^1^Chloroform (CF) and 9,10-anthraquinone (AQ) were applied to the concentrate and partial mixed ration diet (PMR) until week 12.*

*^2^Abundant bacterial phyla were defined as organisms with an average proportion ≥0.05%.*

*^3^Sampling times were from 2 to 49 weeks. Calves during the rearing time were fed as follows: milk twice a day and *ad libitum* control and treatment concentrates at weeks 2 and 4; milk once a day and *ad libitum* control and treatment concentrates and PMR diet at weeks 6, 8 and 10; concentrates step-down weaned and PMR diets fed *ad libitum* until week 14; and grazing a mixed sward of ryegrass/clover as one mob at weeks 24 and 49.*

*^4^A repeated measurement analysis was carried out to determine the effect of methane inhibitors on the bacteria community structure at the phylum level and their carry-over effects.*

*Environmental bacterial phylum.

At the genus level, 41 bacteria genera had a relative abundance ≥0.50% across sampling times. These 41 genera accounted for 85.7 ± 5.38% (mean ± S.D.) of the community across sampling times. On average, *Prevotella* 1 was the most abundant genus (24.7 ± 11.23%), followed by *Christensenellaceae* R-7 group (5.2 ± 5.54%), *Rikenellaceae* RC9 gut group (4.3 ± 3.87%), *Ruminococcus* 2 (4.1 ± 5.61%) and *Sharpea* (3.2 ± 6.54%). The abundant bacteria genera for treated and control calves during different sampling times are shown in Table 6. Treatment by time interaction effects (*P* ≤ 0.048) were observed in 15 abundant bacteria genera; however, pairwise comparison adjusted to Benjamini–Hochberg (BH) showed significance (*P* ≤ 0.040) in only 11 abundant bacteria genera for treated when compared to control calves at random weeks during and 2 weeks after treatment. Treatment effects (*P* < 0.031) were observed in 9 of the 41 abundant bacteria genera. Samples from inhibitor-treated calves indicated proportional increases of *Rikenellaceae* RC9 gut group, *Succiniclasticum*, *Lachnospiraceae* NK3A20 group, *Ruminococcaceae* UCG 002 and p-2534-18B5 gut group, whilst decreases of *Ruminococcus* 1, *Ruminococcaceae* NK4A214 group, *Ruminoclostridium* 5 and *Ruminococcaceae* UCG 005 when compared to control calves. Most of the abundant bacterial genera were affected by time (*P* ≤ 0.029), except for *Succinivibrionaceae* UCG 002, *Ruminiclostridium* 5 and *Eubacterium ventriosum* group (*P* ≥ 0.261).

**TABLE 6:** Effect of methane inhibitors^1^ on the relative abundance of bacterial genus (%)^2^ at different sampling times^3^.

		Time (week)									*P*-value		
Taxa- Genus	Treatment	2	4	6	8	10	14	24	49	SED	P-Tx	P-Tm	P-Int
Prevotella 1	Ctrl	18.36	19.61	20.21	19.24	18.14	18.93	28.10	39.13	4.280	0.945	<0.0001	0.007
	Trt	21.82	27.44	13.03	11.71	23.91	19.84	29.09	39.91				
Christensenellaceae R-7 group	Ctrl	0.34	1.00	4.44	4.63	5.38	4.56	3.79	5.43	0.780	0.608	<0.0001	0.544
	Trt	0.33	0.57	3.85	8.38	6.37	8.41	4.46	5.26				
Rikenellaceae RC9 gut group	Ctrl	**0.13**	0.99	2.04	3.14	2.75	4.13	2.04	4.64	0.637	0.007	<0.0001	0.515
	Trt	**0.55**	1.93	5.32	6.12	5.02	9.31	2.24	4.53				
Ruminococcus 2	Ctrl	2.62	1.19	1.91	2.85	4.22	3.87	0.43	0.27	0.354	0.074	<0.0001	0.288
	Trt	0.52	0.41	4.25	1.99	3.24	2.03	0.44	0.25				
Sharpea	Ctrl	3.55	1.41	0.61	0.53	1.79	0.07	0.01	0.01	0.231	0.644	<0.0001	0.855
	Trt	5.46	2.18	0.93	0.92	2.14	0.26	0.00	0.01				
Bacteroidales BS11 gut group	Ctrl	0.13	1.59	2.95	2.25	1.69	3.05	1.62	2.49	0.324	0.392	<0.0001	0.846
	Trt	0.09	1.08	1.23	2.16	1.49	1.31	1.76	3.02				
Bacteroidales S24-7 group	Ctrl	0.09	0.62	2.80	4.44	2.08	1.05	1.96	1.81	0.333	0.876	<0.0001	0.281
	Trt	0.11	0.29	2.05	1.90	2.52	2.43	2.11	2.39				
Ruminobacter	Ctrl	0.02	0.04	**0.02**	1.18	2.43	0.42	0.02	0.03	0.079	0.363	<0.0001	0.016
	Trt	0.03	0.13	**0.85**	0.62	0.66	0.32	0.02	0.03				
Ruminiclostridium 9	Ctrl	0.03	0.05	0.17	0.20	0.35	2.42	5.86	0.32	0.204	0.508	<0.0001	0.662
	Trt	0.04	0.06	0.20	0.45	1.03	1.59	4.54	0.28				
Lachnospiraceae UCG 005	Ctrl	0.39	0.37	**0.02**	0.02	0.01	0.01	0.01	0.03	0.018	0.484	<0.0001	0.017
	Trt	0.24	0.09	**0.21**	0.07	0.01	0.01	0.01	0.02				
Treponema 2	Ctrl	0.02	0.09	0.88	2.00	0.96	0.43	0.65	0.72	0.153	0.284	<0.0001	0.867
	Trt	0.06	0.21	1.42	1.59	1.76	0.81	0.76	0.77				
Roseburia	Ctrl	2.05	0.82	**0.23**	0.06	0.07	0.24	1.48	0.46	0.171	0.081	<0.0001	0.001
	Trt	4.08	2.35	**1.25**	0.15	0.14	0.11	0.85	0.41				
p-2534-18B5 gut group	Ctrl	0.01	0.01	**0.02**	0.19	0.23	**0.07**	0.10	0.02	0.100	0.000	<0.0001	<0.0001
	Trt	0.01	0.04	**5.13**	1.41	0.53	**0.74**	0.11	0.02				
Lachnospiraceae NK3A20 group	Ctrl	0.01	**0.02**	**0.08**	**0.36**	1.23	1.70	1.22	1.14	0.186	0.001	<0.0001	0.010
	Trt	0.02	**0.18**	**1.09**	**1.76**	1.61	3.28	1.23	1.13				
Succinivibrio	Ctrl	0.68	0.77	0.03	0.05	0.08	0.02	0.00	0.02	0.050	0.604	<0.0001	0.526
	Trt	1.96	0.55	0.03	0.01	0.06	0.01	0.00	0.02				
Succiniclasticum	Ctrl	0.21	1.09	0.76	1.51	0.81	0.65	0.98	1.56	0.237	0.004	<0.0001	0.151
	Trt	0.70	2.47	2.24	1.82	1.99	1.07	1.10	1.47				
Ruminococcus 1	Ctrl	0.45	0.60	1.18	1.44	1.74	1.85	3.17	1.65	0.247	0.009	<0.0001	0.666
	Trt	0.18	0.37	0.70	1.01	1.36	1.42	2.44	1.71				
Ruminococcaceae UCG 014	Ctrl	0.35	0.43	0.85	1.07	1.53	1.77	1.23	0.97	0.171	0.167	<0.0001	0.827
	Trt	0.14	0.29	0.80	1.30	0.97	0.95	1.11	0.93				
Succinivibrionaceae UCG 002	Ctrl	0.06	**0.03**	0.03	0.10	0.06	0.09	0.05	0.07	0.018	0.615	0.261	0.011
	Trt	0.02	**0.64**	0.11	0.06	0.09	0.04	0.02	0.07				
Ruminococcaceae NK4A214 group	Ctrl	**0.36**	0.27	0.82	0.86	0.91	0.76	2.75	2.38	0.197	0.031	<0.0001	0.044
	Trt	**0.09**	0.31	0.58	0.63	0.57	0.63	2.52	2.48				
Ruminiclostridium 5	Ctrl	0.31	**0.93**	**1.35**	**0.77**	0.39	**0.99**	0.28	0.24	0.076	<0.0001	0.273	0.001
	Trt	0.08	**0.04**	**0.08**	**0.11**	0.18	**0.22**	0.32	0.24				
Prevotellaceae UCG 001	Ctrl	0.06	0.48	0.65	0.45	0.51	0.95	0.89	1.67	0.125	0.630	<0.0001	0.034
	Trt	0.23	0.51	0.24	0.28	0.39	0.33	1.05	2.12				
Prevotellaceae UCG 003	Ctrl	0.07	0.05	0.39	0.49	1.49	1.14	0.87	2.60	0.150	0.676	<0.0001	0.105
	Trt	0.07	0.16	0.34	0.46	0.49	0.60	1.26	2.48				
Selenomonas 1	Ctrl	0.27	0.23	0.16	0.10	0.16	0.29	3.41	0.91	0.130	0.059	<0.0001	0.294
	Trt	0.50	0.35	0.12	0.23	0.39	0.35	2.87	0.87				
Fibrobacter	Ctrl	0.01	0.04	0.22	0.27	0.76	0.65	0.39	1.03	0.081	0.751	<0.0001	0.048
	Trt	0.03	0.09	0.04	0.17	0.86	0.21	0.58	1.63				
Pseudobutyrivibrio	Ctrl	0.13	0.16	0.15	0.19	0.16	0.75	1.93	1.42	0.115	0.559	<0.0001	0.869
	Trt	0.19	0.14	0.15	0.29	0.27	0.53	2.07	1.41				
Mollicutes RF9	Ctrl	0.04	0.17	0.26	0.32	0.60	1.22	1.12	0.88	0.119	0.106	<0.0001	0.985
	Trt	0.05	0.25	0.28	0.55	0.76	1.79	1.13	0.84				
Prevotella 7	Ctrl	0.68	0.10	0.01	0.01	0.01	0.01	0.44	0.03	0.027	0.771	<0.0001	0.755
	Trt	0.54	0.21	0.03	0.01	0.01	0.01	0.23	0.03				
Kandleria	Ctrl	0.00	0.00	0.01	0.02	0.07	0.02	1.93	0.03	0.047	0.985	<0.0001	0.759
	Trt	0.00	0.00	0.00	0.01	0.02	0.03	1.83	0.03				
Succinimonas	Ctrl	0.02	0.02	0.02	0.02	0.02	0.01	1.00	0.01	0.027	0.436	<0.0001	0.824
	Trt	0.09	0.02	0.02	0.03	0.04	0.02	1.00	0.01				
Ruminococcaceae UCG 005	Ctrl	0.15	0.19	**0.57**	0.70	0.43	0.62	0.53	0.58	0.075	0.015	<0.0001	0.291
	Trt	0.06	0.09	**0.16**	0.54	0.52	0.35	0.57	0.45				
Eubacterium coprostanoligenes group	Ctrl	0.03	0.12	0.52	0.70	0.71	1.03	0.96	0.91	0.100	0.136	<0.0001	0.417
	Trt	0.03	0.10	0.28	0.38	0.37	0.53	1.04	0.91				
Erysipelotrichaceae UCG 002	Ctrl	0.00	0.01	0.03	0.01	0.05	0.01	0.05	0.00	0.004	0.884	0.002	0.116
	Trt	0.00	0.00	0.01	0.01	0.01	0.09	0.02	0.00				
Atopobium	Ctrl	0.41	0.19	0.28	0.35	0.39	0.51	0.30	0.32	0.060	0.254	0.015	0.036
	Trt	0.13	0.30	0.09	0.40	0.67	0.29	0.31	0.21				
Lachnospiraceae uncultured	Ctrl	0.15	0.14	0.57	0.20	0.15	0.17	0.21	0.25	0.043	0.482	0.029	0.172
	Trt	0.18	0.15	0.23	0.33	0.22	0.30	0.21	0.26				
Prevotellaceae NK3B31 group	Ctrl	**0.01**	0.05	0.04	0.02	0.08	0.09	0.20	0.27	0.024	0.275	0.001	0.002
	Trt	**0.17**	0.43	0.19	0.05	0.04	0.02	0.19	0.23				
Sphaerochaeta	Ctrl	0.10	0.23	0.34	0.66	0.29	0.18	0.09	0.04	0.042	0.764	<0.0001	0.572
	Trt	0.19	0.29	0.21	0.30	0.44	0.21	0.06	0.03				
Lachnospiraceae NK4A136 group	Ctrl	0.05	0.18	0.14	0.20	0.29	0.51	0.60	0.38	0.059	0.338	<0.0001	0.584
	Trt	0.10	0.14	0.40	0.29	0.33	0.67	0.46	0.36				
Ruminococcaceae UCG 002	Ctrl	**0.04**	0.19	0.36	0.27	0.25	0.33	0.49	0.37	0.073	0.001	<0.0001	0.033
	Trt	**0.25**	0.37	0.68	0.66	0.52	0.76	0.38	0.34				
Bacteroidales RF16 group	Ctrl	0.01	0.01	0.03	0.25	**0.47**	**0.85**	0.57	1.28	0.070	0.073	<0.0001	0.000
	Trt	0.01	0.02	0.13	0.18	**0.10**	**0.11**	0.66	1.33				
Eubacterium ventriosum group	Ctrl	0.02	0.04	0.06	0.05	0.04	0.05	0.04	0.06	0.007	0.092	0.274	0.714
	Trt	0.02	0.01	0.01	0.02	0.02	0.04	0.04	0.05				

*Results^4^ are natural log back transformed means and standard error of the differences (SED), P-value for treatment (P-Tx), time (P-Tm) and interaction (P-Int). Significance of pairwise comparisons (Benjamini–Hochberg test) between treatments are shown in bold at each sampling time.*

*^1^Calves were arranged in a control (Ctrl) and treatment (Trt) group. Chloroform (CF) and 9,10-anthraquinone (AQ) were applied to treated partial mixed ration diet (PMR) until week 12.*

*^2^Abundant bacterial genera were defined as organisms with an average proportion ≥0.5%.*

*^3^Sampling times were from 2 to 49 weeks. Calves during the rearing time were fed as follows: milk twice a day and ad libitum control and treatment concentrates at weeks 2 and 4; milk once a day and ad libitum control and treatment concentrates and PMR diet at weeks 6, 8 and 10; concentrates step-down weaned and PMR diets fed ad libitum until week 14; and grazing a mixed sward of ryegrass/clover as one mob at weeks 24 and 49.*

*^4^A repeated measurement analysis was carried out to determine the effect of methane inhibitors on the bacteria community structure at the genus level and their carry-over effects.*

### Archaeal Community

The archaeal community was represented by 17 species at a cut-off of ≥200 amplicons across sampling times. The archaeal community of treated calves had a reduced number of amplicons during and 2 weeks after administration of methane inhibitors compared to control calves (Supplementary Figure 1). The effect of methanogen inhibitors on the abundant archaea species is shown in Table 7. Treatment by time interaction effects (*P* ≤ 0.011) were observed during and 2 weeks after the intake of methane inhibitors. *Post hoc* analyses of *Methanomassiliicoccales* (*Mcc.*) Group 12 sp. ISO4-H5 showed greater (*P* < 0.001; between 6- and 68-fold increases) relative abundance from 2 to 10 weeks, and *Methanosphaera* (*Msp.*) sp. ISO3-F5 showed lower (*P* < 0.001) abundance at weeks 6 and 14 (12 and 31-fold decrease, respectively), in treated when compared to control calves. *Methanobrevibacter (Mbb.) ruminantium* and *Mmc.* Group 4 sp. MpT1 had greater (*P* < 0.001) proportions in treated calves in week 2 (10- and 8-fold change, respectively); however, *Mmc.* Group 4 sp. MpT1 showed significant decreases (*P* < 0.001) in treated calves at weeks 6, 8 and 14 (between 9 and 57-fold reductions) when compared to control calves. Treatment effects showed greater proportions (*P* < 0.001) of *Mmc*. Group 12 sp. ISO4-H5 (2.9% [C.I. = 1.71 – 4.87] vs. 0.4% [C.I. = 0.25 – 0.72]), but lower proportions of *Mbb. gottschalkii* (*P* = 0.028; 34.0% [C.I. = 26.66 – 43.29] vs. 50.8% [C.I. = 40.01 - 64.49]) and *Msp.* sp. ISO3-F5 (*P* = 0.004; 1.0% [C.I. = 0.59 – 1.75] vs. 3.5% [C.I. = 2.12 – 5.80]) in treated when compared to control calves. At weeks 24 and 49, the archaeal groups did not differ between treatments, and *Mmc*. Group 12 sp. ISO4-H5 was almost imperceptible in the rumen of both groups of calves. Time effects (*P* ≤ 0.016) were observed across all the abundant archaea species, except for *Mbb. gottschalkii* whose relative abundance did not change (*P* = 0.061) with time.

**TABLE 7:** Effect of methane inhibitors^1^ on the relative abundance of archaeal species (%)^2^ at different sampling times^3^.

Relative abundance		Time (week)									*P*-value		
Taxa- Genus	Treatment	2	4	6	8	10	14	24	49	SED	P-Tx	P-Tm	P-Int
Methanobrevibacter gottschalkii clade	Ctrl	46.52	49.71	**52.03**	45.77	46.54	59.39	59.89	48.63	15.849	0.028	0.061	0.183
	Trt	31.59	45.54	**14.89**	21.23	36.53	40.96	56.46	46.19				
Methanobrevibacter ruminantium clade	Ctrl	**2.75**	4.55	4.80	13.07	16.97	10.05	15.56	11.72	3.943	0.843	0.016	0.011
	Trt	**26.58**	7.74	2.74	7.06	7.37	3.81	23.07	16.78				
Methanomassiliicoccales Group12 sp. ISO4-H5	Ctrl	**0.11**	**0.33**	**1.63**	**0.96**	**1.36**	1.51	0.07	0.13	1.460	<0.001	<0.001	<0.001
	Trt	**7.48**	**7.52**	**15.37**	**15.02**	**8.22**	4.72	0.06	0.17				
Methanosphaera sp. ISO3-F5	Ctrl	2.03	2.99	**2.73**	1.73	3.04	**2.18**	7.01	16.97	1.531	0.004	<0.001	<0.001
	Trt	1.03	0.98	**0.22**	0.68	0.67	**0.07**	8.91	16.51				
Methanomassiliicoccales Group10 sp.	Ctrl	0.05	0.10	0.02	0.07	0.12	0.15	1.48	9.08	0.470	0.397	<0.001	0.137
	Trt	0.65	0.09	0.06	0.07	0.15	0.03	0.72	7.96				
Methanosphaera sp. Group5	Ctrl	0.13	0.21	0.11	0.06	0.11	0.07	2.24	2.46	0.281	0.202	<0.001	0.569
	Trt	0.54	1.66	0.05	0.05	0.19	0.02	2.80	1.73				
Methanomassiliicoccales Group4 sp. MpT1	Ctrl	**0.19**	0.39	**0.38**	**0.51**	0.81	**1.14**	0.09	0.81	0.199	0.051	<0.001	<0.001
	Trt	**1.53**	0.53	**0.01**	**0.06**	1.24	**0.02**	0.12	0.96				

*Results^4^ are natural log back transformed means and standard error of the differences (SED), P-value for treatment (P-Tx), time (P-Tm) and interaction (P-Int). Significance of pairwise comparisons (Benjamini–Hochberg test) between treatments are shown in bold at each sampling time.*

*^1^Calves were arranged in a control (Ctrl) and treatment (Trt) group. Chloroform (CF) and 9,10-anthraquinone (AQ) were applied to treated partial mixed ration diet (PMR) until week 12.*

*^2^Abundant archaeal species were defined as organisms with an average proportion ≥1.0%.*

*^3^Sampling times were from 2 to 49 weeks. Calves during the rearing time were fed as follows: milk twice a day and ad libitum control and treatment concentrates at weeks 2 and 4; milk once a day and ad libitum control and treatment concentrates and PMR diet at weeks 6, 8 and 10; concentrates step-down weaned and PMR diets fed ad libitum until week 14; and grazing a mixed sward of ryegrass/clover as one mob at weeks 24 and 49.*

*^4^A repeated measurement analysis was carried out to determine the effect of methane inhibitors on the archaeal community structure at the species level and their carry-over effect.*

## Discussion

This study utilized known CH_4_ inhibitors that were incorporated into the solid diet of calves up to 12 weeks of age to investigate the impact on the rumen microbial composition, fermentation profiles, enteric emissions and animal feed intake and growth. In the present study, the inclusion of a CF/AQ mix in the PMR reduced the intake of this diet, but it did not affect the total DMI of solid feeds (concentrate and PMR) of treated calves, resulting in similar growth to control calves during the treatment interventions. Observed results from the current study supported those reported in adult ruminants that showed no effects on feed intake and animal production when receiving comparable doses of halogenated or synthetic compounds to reduce CH_4_ emissions (Sawyer et al., 1974; Martínez-Fernández et al., 2014; Hristov et al., 2015; Martinez-Fernandez et al., 2016, 2018). However, negative dose effects have been indicated in sheep fed up to 66 mg/kg of AQ in the diet for 8 weeks, showing reductions of 19% in DMI and numerical decreases of up to 31% in ADG when compared to controls (Kung et al., 2003). Calves’ LW, ADG, and DMI during the intake of CH_4_ inhibitors in this study were in accordance with those observed in goat kids drenched with bromochloromethane (BCM; Abecia et al., 2013) and calves receiving 3-nitrooxypropanol (3-NOP; Meale et al., 2021) in which DMI and growth were not affected; however, treated goat kids had greater weight gains than their control counterparts, whilst no such effect was observed in the present calf study. Post-weaning, the growth check observed following removal of the starter concentrate over a 2 week period from week 14 to 16 is consistent with prior observations in artificially reared lambs (Jensen et al., 2017). These results suggest that a 2 week transition period to remove starter concentrates provided *ad libitum* to young ruminants is insufficient to enable the rumen and metabolic system of the animal to adapt to the diet change. Therefore, the use of CF/AQ mix in the starter concentrate diets of calves during rumen development can be used without detrimental effects on DMI and growth in the animal; however, further digestibility studies are required to assess the effect that a CF/AQ mix has when used in PMR diet and the post-treatment growth effects on calves of similar breed.

Feeding a CF/AQ mix in the solid feed to calves in the present study showed similar pattern on enteric emissions as reported using CF in dairy cows and steers (Knight et al., 2011; Martinez-Fernandez et al., 2016), and using AQ in sheep (Kung et al., 2003), which resulted in decreasing CH_4_ and increasing H_2_ emissions during treatment but with no differences detected 7 and 14 days post-treatment. Hydrogen disposal, via molecular H_2_, is an important part of the adaptive changes in fermentation pathways in treated calves. In total, molecular H_2_ accounted for 26% of the H_2_ that was not captured in methane. This proportion of molecular H_2_ is in accordance with the 15 to 30% value observed by Martinez-Fernandez et al. (2016) in adult cattle. In response to CH_4_ inhibition, the proportion of propionate was increased in the current trial. The propionate fermentation pathway consumes H_2_ and is in direct competition with methanogenesis (Iannotti et al., 1973; Wolin, 1976; Morvan et al., 1996). However, because only rumen concentrations in this experiment were measured, no quantitative estimate of the H_2_ captured in propionate could be made. Following methane inhibition, the observed increases in ruminal H_2_ in treated calves additionally enhanced the fermentation pathways that consume H_2_ such as valerate and caproate, as also indicated in a meta-analysis study by Ungerfeld (2015). It has been indicated that H_2_ is redirected toward the production of SCFA that require a net incorporation of H_2_ when produced from glucose, i.e., production of valerate and caproate via propionyl-CoA, or SCFA whose production results in less release of H_2_ per unit of glucose compared to acetate, i.e., caproate via acetyl-CoA/butyryl-CoA (Ungerfeld, 2020). Acetogenesis is another potential pathway of H_2_ disposal in the rumen, but can be excluded here since CF inhibits the acetyl-CoA cleavage pathway of acetogens (Scholten et al., 2000) although no measurements of the pathway were made in this experiment.

In ruminants, methanogens have been found in neonatal animals (Guzman et al., 2015), indicating that microbial colonization of the gastrointestinal tract (GIT) occurred during or directly after birth (Malmuthuge and Griebel, 2018). In the current study, calves entered the experiment at approximately 4 days of age, therefore these animals had already been exposed to maternal and environmental microbial communities. Exposing the community to increasing amounts of CF/AQ, during the first 12 weeks of rearing, affected the diversity of the archaea community acquired during the first 4 days of the calves’ life. However, the observed changes of the archaeal community diversity were not maintained, returning to control levels 12 weeks after treatment cessation. Similar observations have been previously reported by Abecia et al. (2013) in goat kids ingesting BCM that showed reductions in the archaeal diversity while ingesting the inhibitors, but 12 weeks after treatment cessation all groups had similar diversities. Our results indicate that the application of CF/AQ in the solid feed during the first 12 weeks of rearing does not lead to a permanent change in the diversity of the archaeal community in growing ruminants.

The composition of the methanogen community in the rumen has been suggested to change from birth to adulthood (Guzman et al., 2015; Friedman et al., 2017). In the present study, changes in the archaeal community corresponded to those reported in calves receiving 3-NOP, in which the archaea diversity increased as the animal aged (Meale et al., 2021). In adult ruminants, the family *Methanobacteriaceae*, which includes *Methanobrevibacter* spp. and *Methanosphaera* spp., represents up to 90% of the rumen archaea (Henderson et al., 2015; Seedorf et al., 2015; Friedman et al., 2017). The relative abundance of this family in control calves showed adult-like proportions, representing an average of 93.8 ± 3.89% of the archaea community between 2 and 49 weeks. Methanogen inhibitors like CF and 3-NOP in steers have been shown to decrease the relative abundance of *Methanobacteriaceae* (Martinez-Fernandez et al., 2018). These results were consistent with the reduction of *Mbb. gottschalkii* and *Msp.* ISO3-F5 found in calves fed CF/AQ. Little treatment by time interaction effects observed between treatment calves for *Methanobacteriaceae* in the present study agreed with those reported in calves receiving 3-NOP from birth until 3 weeks post-weaning by Meale et al. (2021). Quantitative PCR analysis confirmed that 3-NOP and CF reduces *Methanobrevibacter*; however, only 3-NOP decreased the abundance of Methanomassiliicoccaceae family (Martinez-Fernandez et al., 2018). The mix of inhibitors in our experiment did not affect Methanomassiliicoccaceae confirming the results from Martinez-Fernandez et al. (2018) and also indicating that AQ does not appear to have a specific effect against this archaea family. The most abundant species of *Methanobrevibacter* grow from reducing H_2_ and CO_2_ (Miller et al., 1986), whilst *Methanosphaera* is a methanogen that reduces methanol with H_2_ and is dependent on acetate as a carbon source (Fricke et al., 2006). However, *Methanomassiliicoccales (Mmc.)* spp. are obligatory hydrogen-dependent methylotrophic methanogens and require compounds like methanol, methylamine, dimethylamine, and trimethylamine as their major energy and carbon source (Lang et al., 2015; Li et al., 2016). In the present study, it is not clear how the use of two different methanogen inhibitors affects the growth and abundance of the different archaea species (Borrel et al., 2014). Additionally, the observed changes in the archaeal community were driven by the diet offered at the time as observed in control calves. In addition, care has to be taken when evaluating the relative abundances, as an increase in abundance of one group can be due to either an increase in the target groups’ numbers or a major decrease in other groups (Supplementary Figure 1).

The use of methanogen inhibitors did not affect the diversity of the bacterial community in the present study. This is a novel observation in young calves and an unexpected result since the rumen metabolite profiles (gases and SCFA) were altered by the CF/AQ mix fed to the calves. The results in the present study agree with those reported in lambs and calves also receiving methane inhibitors from birth until weaning off milk and 3 weeks after weaning off milk, respectively, in which alterations of the fermentation pathways did not affect the bacterial diversity in the rumen when compared to controls (Abecia et al., 2018; Meale et al., 2021). Bacterial community diversity increased over time in the present study. Similar results have been shown for growing calves in previous studies (Jami et al., 2013; Dill-McFarland et al., 2017; Meale et al., 2021). In addition to animal age, bacterial community diversity can be affected by the diet (Kim et al., 2016; Martinez-Fernandez et al., 2016). In our study, the effects of time on bacteria community diversity reported here are a combination of animal age and changing diet.

Despite the differences in intra-ruminal H_2_ concentrations observed between treatment groups in the present study, the bacterial community at the phylum level was similar between studied groups. Our results differed from those in cannulated steers where increases in relative abundance of *Bacteroidetes* and decreases in *Firmicutes* in response to CF were described (Martinez-Fernandez et al., 2016). Changes in the abundant bacterial genera were few notwithstanding the fermentation shifts in the concentration of the different SCFA observed in the rumens of treated compared to control calves. The increase of *Lachnospiraceae* NK3A20 observed in treated calves was in accordance with data from CH_4_ inhibition studies in goat kids supplemented with rhubarb root containing emodin, a derivative of AQ (Wang et al., 2017). This family possess a large and diverse repertoire of glycoside hydrolases and polysaccharide lyases (Seshadri et al., 2018), having the capacity to ferment polysaccharides or fumarate to acetate, succinate and CO_2_, while no H_2_ is formed (Janssen and Hugenholtz, 2003). In the present study, CH_4_ inhibition appeared to increase *Rikenellaceae* RC9 gut group, *Roseburia* and p-2534-18B5 gut group to different extents. This is similar to the observation where these bacteria groups were increased by the ingestion of concentrate diets (Henderson et al., 2015), in the sense that diets rich in grains are known to lead to lower CH_4_ emissions, because starch utilizing bacteria tend to produce less H_2_ (Stewart et al., 1997; Janssen, 2010). Therefore, the metabolism of these bacteria is not likely to be affected by the partial pressure of H_2_ in the rumen.

Cellulolytic microbes such as *Fibrobacter* were not affected by CH_4_ inhibition in the present study, which is in accordance with previous observations inhibiting CH_4_ production in steers dosed with CF (16 and 26 mg/kg of LW) (Martinez-Fernandez et al., 2016) and in steers receiving CF (16 mg/kg of LW) or 3-NOP (2.5 g/animal) (Martinez-Fernandez et al., 2018). These results confirm that *Fibrobacter* can tolerate H_2_ accumulation (Wolin et al., 1997) because its major end product is succinate (Abdul Rahman et al., 2016). In contrast, calves treated with CF/AQ mixed in the diet showed that the proportion of cellulolytic *Ruminiclostridium* and *Ruminococcus* was reduced with increased H_2_ concentrations, similar to observations with BCM (50 mg/kg LW) in the diet (Mitsumori et al., 2012). Inhibition of these cellulolytic bacteria genera has been observed previously during *in vitro* experiments, in which inhibition of CH_4_ production with haloforms reduced *Ruminococcus* populations, whilst *Fibrobacter* numbers increased (Goel et al., 2009). The class *Clostridia* is predominated by H_2_-producing cellulolytic bacteria, and the H_2_ accumulation significantly inhibited their H_2_-producing activity (Lay, 2001). The increased partial pressure of H_2_ influences the metabolism of these fiber-degrading genera by inhibiting NADH oxidation, whilst H_2_ is diverted to form other end products such as succinate and ethanol (Wolin et al., 1997). Therefore, the degradation of cellulose by these *Ruminiclostridium* and *Ruminococcus* (Ruminococcaceae family) may be impaired by the increased H_2_ pressure in the rumen.

Methane inhibition did not have any effect on the most abundant genus *Prevotella* capable of degrading a broad spectrum of polysaccharides and peptides in the diet. They also have the ability to use different pathways in response to H_2_ pressure which makes them very flexible under high H_2_ conditions (Marounek and Dušková, 1999; Seshadri et al., 2018) as induced in the treated calves. Given the minor changes observed in the rumen community composition, this indicates that methane inhibition, other than dietary changes, result predominantly in a shift of metabolic pathways of an existing community rather than a change in the microbial communities. Further investigations are necessary to elucidate the mechanisms that the rumen bacterial population uses to adapt to high intra-ruminal H_2_ pressure, produced by the inhibition of methanogen microbes with CF/AQ in the diet, and to explore whether imprinting of the rumen microbiota is possible.

## Conclusion

Collectively, this study showed that CF/AQ mix inclusion in the starter diets during the first 12 weeks of rearing did not affect the DMI and growth of dairy calves. Methane inhibition changed the composition of the target community, the methanogens, but had only minor effects on the bacterial community indicating the importance of the metabolic flexibility of many rumen microorganisms. This metabolic flexibility, however, may account for the observation that there was no lasting effect of the microbial community as it returns to the energetically more favorable pathways once CH_4_ inhibition/H_2_ pressure has been removed. Metabolic flexibility of the rumen microbiota, however, may be overcome if the treatments begin earlier in the rumen and more work is needed to establish the start and the duration of such early life interventions.

## Data Availability Statement

The data presented in the study are deposited in the European Nucleotide Archive, accession number PRJEB37781.

## Ethics Statement

The animal study was reviewed and approved by Grasslands Animal Ethics Committee.

## Author Contributions

SM designed the study, secured funding, and generated the respiration chamber data. OC-C generated the microbial community data and wrote the initial draft manuscript. SG and OC-C undertook the statistical analysis. All authors contributed to the data interpretation and manuscript editing.

## Conflict of Interest

The authors declare that the research was conducted in the absence of any commercial or financial relationships that could be construed as a potential conflict of interest.

## Publisher’s Note

All claims expressed in this article are solely those of the authors and do not necessarily represent those of their affiliated organizations, or those of the publisher, the editors and the reviewers. Any product that may be evaluated in this article, or claim that may be made by its manufacturer, is not guaranteed or endorsed by the publisher.
