# Supplementary material for: Effect of Methane Inhibitors on Ruminal Microbiota During Early Life and Its Relationship With Ruminal Metabolism and Growth in Calves
Source: Front Microbiol. 2021 Sep 16;12:710914. doi: 10.3389/fmicb.2021.710914 (PMC8482044; doi:10.3389/fmicb.2021.710914)

Supplementary Figures

Supplementary Figure 1. Color-coded bar plot showing the archaea amplicons as a function of interventions modulating the rumen methanogens in control (Ctrl) and treatment (Trt) calves during different sampling times. Data shown in columns correspond to the number of total Illumina read numbers for each OTU identified (Y axis) within each group of calves at different times of rearing (X axis). Calves are arranged in a control (Ctrl) and treatment (Trt) group. Chloroform (CF) and 9,10-anthraquinone (AQ) were applied to the concentrate and total mixed ration diet (PMR) until week 12. Calves during the rearing time in both groups were fed as follows: milk twice a day and *ad libitum* concentrate diets at weeks 2 and 4; milk once a day and *ad libitum* concentrates and PMR diets at weeks 6, 8 and 10; concentrates step-down weaned and PMR diets fed *ad libitum* until week 14; and grazing a mixed sward of ryegrass/clover as one mob at weeks 24 and 49.

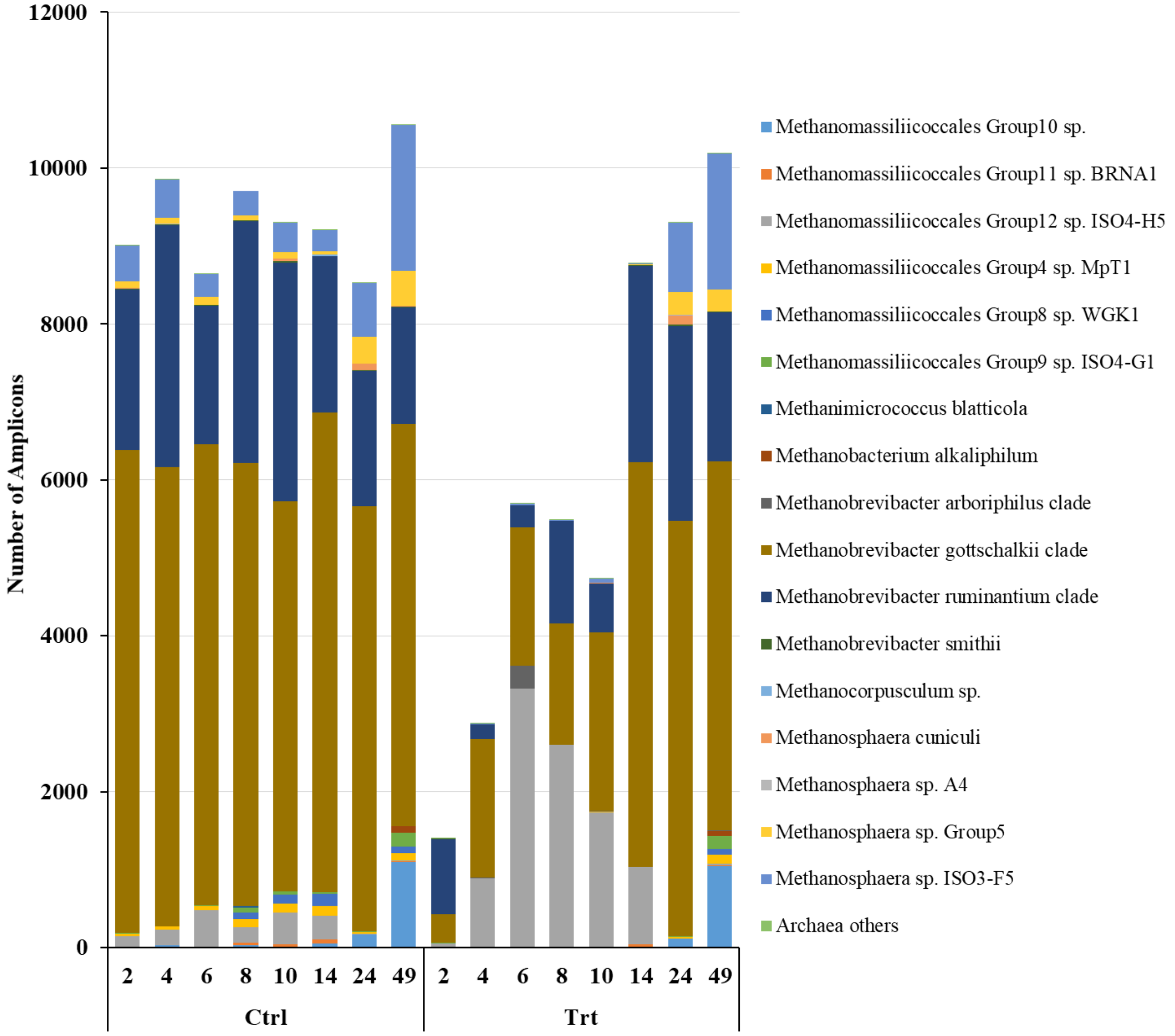

Supplement: Supplementary file 1 [file Image_1.pdf]
