# Supplementary material for: Effect of Methane Inhibitors on Ruminal Microbiota During Early Life and Its Relationship With Ruminal Metabolism and Growth in Calves
Source: Front Microbiol. 2021 Sep 16;12:710914. doi: 10.3389/fmicb.2021.710914 (PMC8482044; doi:10.3389/fmicb.2021.710914)
Supplement: Supplementary file 2 [file Table_1.pdf]

Supplementary Table 1. Sequencing primers used to target the 16S rRNA gene.

Name	Direction	Target	5'-3' Sequence
Ba9F	Forward	Universal bacteria	GAGTTTGATCMTGGCTCAG
Ba515Rmod1	Reverse		CCGCGGCKGCTGGCAC
Ar915aF	Forward	Universal archaea	AGGAATTGGCGGGGGAGCAC
Ar1386R	Reverse		GCGGTGTGTGCAAGGAGC
