# Supplementary material for: Effect of Methane Inhibitors on Ruminal Microbiota During Early Life and Its Relationship With Ruminal Metabolism and Growth in Calves
Source: Front Microbiol. 2021 Sep 16;12:710914. doi: 10.3389/fmicb.2021.710914 (PMC8482044; doi:10.3389/fmicb.2021.710914)
Supplement: Supplementary file 3 [file Table_2.pdf]

Supplementary Table 2. Phyla composition of the ruminal bacteria in control (Ctrl) and treated (Trt) calved across the different sampling times (weeks) of rearing. Bacterial phyla highlighted in bold are the most abundant in the rumen of calves.

**Time (weeks)**	**2**		**4**		**6**		**8**		**10**		**14**		**24**		**49**	
**Treatment**	**Ctr**	**Trt**	**Ctrl**	**Trt**	**Ctrl**	**Trt**	**Ctrl**	**Trt**	**Ctrl**	**Trt**	**Ctrl**	**Trt**	**Ctrl**	**Trt**	**Ctrl**	**Trt**
**Firmicutes**	48.13	41.12	40.47	31.18	43.07	46.95	38.34	49.42	47.37	42.83	49.16	45.11	53.60	51.26	31.60	30.49
**Bacteroidetes**	39.62	39.18	42.67	46.80	39.38	38.26	40.38	37.53	37.08	43.84	39.36	43.46	40.54	42.59	59.10	59.89
**Proteobacteria**	9.92	16.89	13.01	16.77	5.04	8.44	11.87	6.36	8.52	4.25	5.05	5.32	0.74	0.57	2.53	2.24
Spirochaetae	0.68	1.24	1.35	2.47	7.81	3.33	6.11	3.06	2.13	3.40	0.89	1.88	1.00	1.08	0.97	1.12
Tenericutes	0.22	0.19	0.40	0.63	0.55	0.79	0.61	1.06	0.79	0.90	1.70	1.97	1.46	1.55	1.35	1.42
Fibrobacteres	0.04	0.15	0.12	0.27	1.19	0.12	0.83	0.84	1.72	2.33	0.91	0.80	0.55	0.91	1.23	1.92
Actinobacteria	1.14	0.96	1.18	1.61	1.02	0.49	0.77	0.76	0.71	1.80	0.66	0.66	0.51	0.57	0.51	0.39
Cyanobacteria	0.03	0.02	0.02	0.01	0.14	0.11	0.24	0.07	0.41	0.08	0.84	0.15	0.42	0.35	0.98	1.00
SHA 109	0.01	0.01	0.23	0.03	0.98	1.16	0.17	0.43	0.58	0.14	0.13	0.05	0.19	0.09	0.05	0.05
Saccharibacteria	0.00	0.00	0.00	0.00	0.09	0.02	0.06	0.09	0.14	0.11	0.11	0.20	0.32	0.44	0.49	0.41
Lentisphaerae	0.02	0.01	0.05	0.02	0.13	0.03	0.15	0.05	0.14	0.04	0.56	0.06	0.10	0.12	0.30	0.27
Elusimicrobia	0.04	0.01	0.29	0.00	0.17	0.01	0.08	0.00	0.09	0.00	0.14	0.01	0.03	0.02	0.04	0.05
Chloroflexi	0.01	0.00	0.05	0.02	0.14	0.03	0.09	0.05	0.07	0.05	0.06	0.03	0.05	0.04	0.08	0.07
Synergistetes	0.02	0.06	0.03	0.05	0.07	0.01	0.07	0.04	0.05	0.01	0.09	0.01	0.02	0.01	0.04	0.04
Candidate division SR1	0.00	0.00	0.00	0.00	0.00	0.00	0.00	0.00	0.00	0.00	0.00	0.03	0.10	0.06	0.20	0.18
Planctomycetes	0.00	0.00	0.01	0.00	0.04	0.03	0.04	0.02	0.02	0.01	0.03	0.02	0.01	0.02	0.04	0.02
Armatimonadetes	0.00	0.00	0.00	0.00	0.00	0.00	0.00	0.00	0.00	0.00	0.01	0.00	0.01	0.01	0.02	0.02
Chlamydiae	0.00	0.00	0.00	0.00	0.00	0.00	0.00	0.00	0.00	0.00	0.02	0.00	0.00	0.00	0.01	0.01
