# Supplementary material for: Effect of Methane Inhibitors on Ruminal Microbiota During Early Life and Its Relationship With Ruminal Metabolism and Growth in Calves
Source: Front Microbiol. 2021 Sep 16;12:710914. doi: 10.3389/fmicb.2021.710914 (PMC8482044; doi:10.3389/fmicb.2021.710914)
Supplement: Supplementary file 4 [file Table_3.pdf]

Supplementary Table 3. Class composition of the ruminal bacteria in control (Ctrl) and treated (Trt) calved across the different sampling times (weeks) of rearing. Bacterial classes highlighted in bold are the most abundant in the rumen of calves.

**Time (weeks)**	**2**		**4**		**6**		**8**		**10**		**14**		**24**		**49**	
**Treatment**	**Ctr**	**Trt**	**Ctr**	**Trt**	**Ctr**	**Trt**	**Ctr**	**Trt**	**Ctr**	**Trt**	**Ctr**	**Trt**	**Ctr**	**Trt**	**Ctr**	**Trt**
**Bacteroidia**	39.62	39.18	42.67	46.80	39.38	38.25	40.36	37.52	37.06	43.82	39.27	43.45	40.41	42.47	58.97	59.81
**Clostridia**	33.62	26.53	31.94	22.06	37.97	41.15	32.45	41.62	33.73	32.31	44.08	37.23	41.49	40.26	27.33	26.80
**Gammaproteobacteria**	9.39	14.78	12.67	15.49	4.60	8.01	11.40	5.88	7.98	3.80	3.85	4.33	0.20	0.13	0.90	0.92
**Erysipelotrichia**	9.07	10.34	5.30	4.43	3.11	2.77	3.65	5.03	11.85	5.47	3.31	4.22	6.38	5.09	1.00	0.78
**Negativicutes**	5.37	4.18	3.19	4.65	1.93	2.89	2.19	2.67	1.51	2.98	1.37	1.95	5.31	5.21	3.13	2.81
**Spirochaetes**	0.68	1.24	1.35	2.47	7.81	3.33	6.11	3.06	2.13	3.40	0.89	1.88	1.00	1.08	0.97	1.12
**Mollicutes**	0.22	0.19	0.40	0.63	0.55	0.79	0.61	1.06	0.79	0.90	1.70	1.97	1.46	1.55	1.35	1.42
**Fibrobacteria**	0.04	0.15	0.12	0.27	1.19	0.12	0.83	0.84	1.72	2.33	0.91	0.80	0.55	0.91	1.23	1.92
Coriobacteriia	1.13	0.95	1.17	1.61	1.00	0.47	0.75	0.74	0.69	1.77	0.63	0.62	0.48	0.54	0.45	0.32
Alphaproteobacteria	0.04	0.01	0.04	0.09	0.28	0.17	0.32	0.36	0.43	0.28	1.11	0.89	0.42	0.30	1.24	0.96
Bacilli	0.07	0.08	0.04	0.04	0.06	0.15	0.05	0.10	0.27	2.08	0.40	1.71	0.42	0.70	0.13	0.10
Deltaproteobacteria	0.32	1.97	0.23	1.08	0.11	0.18	0.09	0.08	0.04	0.08	0.05	0.07	0.07	0.07	0.20	0.17
Melainabacteria	0.03	0.01	0.02	0.01	0.14	0.11	0.23	0.06	0.40	0.07	0.82	0.13	0.36	0.30	0.98	1.00
SHA 109 *	0.01	0.01	0.23	0.03	0.98	1.16	0.17	0.43	0.58	0.14	0.13	0.05	0.19	0.09	0.05	0.05
Saccharibacteria *	0.00	0.00	0.00	0.00	0.09	0.02	0.06	0.09	0.14	0.11	0.11	0.20	0.32	0.44	0.49	0.41
Betaproteobacteria	0.08	0.08	0.04	0.06	0.03	0.08	0.04	0.04	0.05	0.08	0.03	0.03	0.04	0.06	0.17	0.20
Lentisphaeria	0.00	0.00	0.00	0.00	0.06	0.00	0.09	0.00	0.09	0.00	0.40	0.00	0.05	0.06	0.13	0.12
Elusimicrobia	0.04	0.01	0.29	0.00	0.17	0.01	0.08	0.00	0.09	0.00	0.14	0.01	0.03	0.02	0.04	0.05
Anaerolineae	0.01	0.00	0.05	0.02	0.14	0.03	0.09	0.05	0.07	0.05	0.06	0.03	0.04	0.04	0.06	0.06
Lentisphaerae RFP12 gut group	0.02	0.00	0.04	0.01	0.06	0.02	0.06	0.05	0.05	0.03	0.12	0.05	0.03	0.03	0.09	0.09
Synergistia	0.02	0.06	0.03	0.05	0.07	0.01	0.07	0.04	0.05	0.01	0.09	0.01	0.02	0.01	0.04	0.04
Candidate division SR1 *	0.00	0.00	0.00	0.00	0.00	0.00	0.00	0.00	0.00	0.00	0.00	0.03	0.10	0.06	0.20	0.18

Actinobacteria	0.01	0.01	0.01	0.01	0.01	0.02	0.02	0.02	0.02	0.03	0.03	0.04	0.03	0.03	0.06	0.07
Bacteroidetes VC2.1 Bac22	0.00	0.00	0.00	0.00	0.00	0.00	0.02	0.00	0.01	0.00	0.01	0.00	0.05	0.07	0.11	0.06
Planctomycetacia	0.00	0.00	0.01	0.00	0.04	0.03	0.04	0.02	0.02	0.01	0.03	0.02	0.01	0.02	0.04	0.02
Epsilonproteobacteria	0.07	0.05	0.03	0.04	0.02	0.01	0.03	0.01	0.02	0.01	0.01	0.00	0.00	0.01	0.01	0.00
Oligosphaeria	0.00	0.00	0.00	0.00	0.00	0.00	0.00	0.00	0.00	0.00	0.04	0.00	0.02	0.03	0.07	0.05
Bacteroidetes BD2-2	0.00	0.00	0.00	0.00	0.00	0.00	0.00	0.00	0.00	0.01	0.07	0.01	0.06	0.03	0.01	0.02
Chloroplast	0.00	0.00	0.00	0.00	0.01	0.01	0.01	0.01	0.01	0.01	0.02	0.01	0.06	0.04	0.01	0.01
Armatimonadetes *	0.00	0.00	0.00	0.00	0.00	0.00	0.00	0.00	0.00	0.00	0.01	0.00	0.01	0.01	0.02	0.02
Flavobacteriia	0.00	0.00	0.00	0.00	0.00	0.01	0.00	0.00	0.01	0.01	0.00	0.00	0.01	0.01	0.00	0.00
Dehalococcoidia	0.00	0.00	0.00	0.00	0.00	0.00	0.00	0.00	0.00	0.00	0.00	0.00	0.02	0.00	0.02	0.01
WCHB1-25	0.00	0.00	0.00	0.01	0.00	0.00	0.00	0.00	0.00	0.00	0.00	0.00	0.00	0.00	0.01	0.00
Chlamydiae	0.00	0.00	0.00	0.00	0.00	0.00	0.00	0.00	0.00	0.00	0.02	0.00	0.00	0.00	0.01	0.01
Other classes	0.14	0.17	0.13	0.14	0.17	0.22	0.19	0.21	0.18	0.20	0.30	0.22	0.35	0.32	0.44	0.41

* Unclassified class
