# Supplementary material for: Effect of Methane Inhibitors on Ruminal Microbiota During Early Life and Its Relationship With Ruminal Metabolism and Growth in Calves
Source: Front Microbiol. 2021 Sep 16;12:710914. doi: 10.3389/fmicb.2021.710914 (PMC8482044; doi:10.3389/fmicb.2021.710914)
Supplement: Supplementary file 5 [file Table_4.pdf]

Supplementary Table 4. Order composition of the ruminal bacteria in control (Ctrl) and treated (Trt) calved across the different sampling times (weeks) of rearing. Bacterial orders highlighted in bold are the most abundant in the rumen of calves.

**Time (weeks)**	**2**		**4**		**6**		**8**		**10**		**14**		**24**		**49**	
**Treatment**	**Ctrl**	**Trt**	**Ctrl**	**Trt**	**Ctrl**	**Trt**	**Ctrl**	**Trt**	**Ctrl**	**Trt**	**Ctrl**	**Trt**	**Ctrl**	**Trt**	**Ctrl**	**Trt**
**Bacteroidales**	39.62	39.18	42.67	46.80	39.38	38.25	40.36	37.52	37.06	43.82	39.27	43.45	40.41	42.47	58.97	59.81
**Clostridiales**	33.62	26.53	31.94	22.06	37.97	41.15	32.45	41.62	33.73	32.31	44.08	37.23	41.49	40.26	27.33	26.80
**Aeromonadales**	9.38	14.75	12.66	15.47	4.58	7.97	11.37	5.83	7.93	3.75	3.82	4.30	0.11	0.07	0.16	0.17
**Erysipelotrichales**	9.07	10.34	5.30	4.43	3.11	2.77	3.65	5.03	11.85	5.47	3.31	4.22	6.38	5.09	1.00	0.78
**Selenomonadales**	5.37	4.18	3.19	4.65	1.93	2.89	2.19	2.67	1.51	2.98	1.37	1.95	5.31	5.21	3.13	2.81
**Spirochaetales**	0.68	1.24	1.35	2.47	7.81	3.33	6.11	3.06	2.13	3.40	0.89	1.88	1.00	1.08	0.97	1.12
**Fibrobacterales**	0.04	0.15	0.12	0.27	1.19	0.12	0.83	0.84	1.72	2.33	0.91	0.80	0.55	0.91	1.23	1.92
**Coriobacteriales**	1.13	0.95	1.17	1.61	1.00	0.47	0.75	0.74	0.69	1.77	0.63	0.62	0.48	0.54	0.45	0.32
**Mollicutes RF9**	0.08	0.16	0.30	0.60	0.39	0.72	0.57	1.02	0.78	0.87	1.50	1.93	1.16	1.21	0.91	0.87
**Lactobacillales**	0.07	0.07	0.04	0.04	0.06	0.15	0.05	0.10	0.27	2.08	0.40	1.70	0.42	0.70	0.12	0.09
**Rhodospirillales**	0.04	0.00	0.04	0.09	0.27	0.15	0.31	0.34	0.41	0.24	1.06	0.81	0.37	0.25	1.01	0.74
**Gastranaerophilales**	0.03	0.01	0.02	0.01	0.14	0.11	0.23	0.06	0.40	0.07	0.82	0.13	0.36	0.30	0.98	1.00
**SHA 109 ***	0.01	0.01	0.23	0.03	0.98	1.16	0.17	0.43	0.58	0.14	0.13	0.05	0.19	0.09	0.05	0.05
**Desulfovibrionales**	0.31	1.97	0.20	1.08	0.05	0.10	0.03	0.04	0.01	0.01	0.01	0.01	0.01	0.02	0.03	0.03
Saccharibacteria *	0.00	0.00	0.00	0.00	0.09	0.02	0.06	0.09	0.14	0.11	0.11	0.20	0.32	0.44	0.49	0.41
Anaeroplasmatales	0.05	0.00	0.01	0.01	0.08	0.04	0.04	0.02	0.01	0.03	0.18	0.04	0.28	0.32	0.39	0.51
Enterobacteriales	0.01	0.00	0.00	0.00	0.00	0.01	0.01	0.01	0.01	0.01	0.00	0.01	0.02	0.01	0.64	0.67
Victivallales	0.00	0.00	0.00	0.00	0.06	0.00	0.09	0.00	0.09	0.00	0.40	0.00	0.05	0.06	0.13	0.12
Anaerolineales	0.01	0.00	0.05	0.02	0.14	0.03	0.09	0.05	0.07	0.05	0.06	0.03	0.04	0.04	0.06	0.06
Lentisphaerae RFP12 gut group *	0.02	0.00	0.04	0.01	0.06	0.02	0.06	0.05	0.05	0.03	0.12	0.05	0.03	0.03	0.09	0.09
Burkholderiales	0.02	0.04	0.01	0.03	0.01	0.05	0.02	0.02	0.03	0.06	0.02	0.02	0.04	0.05	0.15	0.17
Desulfuromonadales	0.01	0.00	0.03	0.00	0.06	0.08	0.06	0.03	0.02	0.06	0.03	0.06	0.06	0.05	0.06	0.09
Lineage I (Endomicrobia)	0.01	0.00	0.28	0.00	0.11	0.00	0.05	0.00	0.06	0.00	0.11	0.00	0.00	0.00	0.01	0.01

Synergistales	0.02	0.06	0.03	0.05	0.07	0.01	0.07	0.04	0.05	0.01	0.09	0.01	0.02	0.01	0.04	0.04
Candidate division SR1 *	0.00	0.00	0.00	0.00	0.00	0.00	0.00	0.00	0.00	0.00	0.00	0.03	0.10	0.06	0.20	0.18
NB1-n	0.06	0.03	0.09	0.02	0.08	0.00	0.01	0.01	0.01	0.00	0.02	0.01	0.02	0.01	0.05	0.04
Rhizobiales	0.00	0.00	0.00	0.00	0.00	0.00	0.01	0.01	0.01	0.02	0.02	0.03	0.02	0.02	0.15	0.15
Bacteroidetes VC2.1 Bac22 *	0.00	0.00	0.00	0.00	0.00	0.00	0.02	0.00	0.01	0.00	0.01	0.00	0.05	0.07	0.11	0.06
Pseudomonadales	0.01	0.01	0.00	0.01	0.01	0.02	0.01	0.02	0.01	0.02	0.01	0.01	0.06	0.03	0.07	0.06
Planctomycetales	0.00	0.00	0.01	0.00	0.04	0.03	0.04	0.02	0.02	0.01	0.03	0.02	0.01	0.02	0.04	0.02
Campylobacterales	0.07	0.05	0.03	0.04	0.02	0.01	0.03	0.01	0.02	0.01	0.01	0.00	0.00	0.01	0.01	0.00
Elusimicrobiales	0.04	0.01	0.00	0.00	0.05	0.01	0.02	0.00	0.03	0.00	0.03	0.01	0.02	0.01	0.03	0.04
Neisseriales	0.06	0.04	0.02	0.03	0.01	0.02	0.02	0.01	0.01	0.02	0.00	0.01	0.01	0.01	0.01	0.01
Corynebacteriales	0.01	0.01	0.01	0.01	0.01	0.01	0.01	0.02	0.01	0.03	0.02	0.02	0.02	0.02	0.04	0.05
Oligosphaerales	0.00	0.00	0.00	0.00	0.00	0.00	0.00	0.00	0.00	0.00	0.04	0.00	0.02	0.03	0.07	0.05
Bacteroidetes BD2-2 *	0.00	0.00	0.00	0.00	0.00	0.00	0.00	0.00	0.00	0.01	0.07	0.01	0.06	0.03	0.01	0.02
Chloroplast *	0.00	0.00	0.00	0.00	0.01	0.01	0.01	0.01	0.01	0.01	0.02	0.01	0.06	0.04	0.01	0.01
Pasteurellales	0.01	0.01	0.01	0.01	0.01	0.01	0.01	0.01	0.02	0.02	0.01	0.01	0.02	0.02	0.03	0.02
Sphingomonadales	0.00	0.00	0.00	0.00	0.00	0.00	0.00	0.00	0.00	0.02	0.02	0.02	0.03	0.03	0.02	0.02
Oligoflexales	0.00	0.00	0.00	0.00	0.00	0.00	0.00	0.00	0.00	0.00	0.00	0.00	0.00	0.00	0.10	0.04
Rickettsiales	0.00	0.01	0.00	0.00	0.00	0.00	0.00	0.00	0.00	0.01	0.01	0.02	0.00	0.00	0.05	0.04
Micrococcales	0.00	0.00	0.00	0.00	0.00	0.01	0.01	0.01	0.00	0.01	0.01	0.02	0.01	0.01	0.02	0.02
Acholeplasmatales	0.03	0.00	0.00	0.00	0.00	0.03	0.00	0.01	0.00	0.00	0.00	0.00	0.00	0.01	0.00	0.00
Armatimonadetes *	0.00	0.00	0.00	0.00	0.00	0.00	0.00	0.00	0.00	0.00	0.01	0.00	0.01	0.01	0.02	0.02
UCT N117	0.00	0.00	0.00	0.00	0.01	0.00	0.01	0.00	0.01	0.00	0.01	0.00	0.00	0.00	0.01	0.02
Flavobacteriales	0.00	0.00	0.00	0.00	0.00	0.01	0.00	0.00	0.01	0.01	0.00	0.00	0.01	0.01	0.00	0.00
Caulobacterales	0.00	0.00	0.00	0.00	0.01	0.01	0.00	0.00	0.01	0.01	0.00	0.01	0.00	0.00	0.00	0.00
MSBL5	0.00	0.00	0.00	0.00	0.00	0.00	0.00	0.00	0.00	0.00	0.00	0.00	0.02	0.00	0.02	0.01
WCHB1-25 *	0.00	0.00	0.00	0.01	0.00	0.00	0.00	0.00	0.00	0.00	0.00	0.00	0.00	0.00	0.01	0.00
Desulfobacterales	0.00	0.00	0.00	0.00	0.00	0.01	0.00	0.00	0.00	0.01	0.00	0.00	0.00	0.00	0.00	0.00
Order Incertae Sedis	0.00	0.00	0.00	0.00	0.00	0.00	0.00	0.01	0.01	0.01	0.01	0.01	0.00	0.00	0.00	0.00

Bacillales	0.00	0.00	0.00	0.00	0.00	0.00	0.00	0.00	0.00	0.00	0.00	0.00	0.00	0.00	0.01	0.02
Chlamydiales	0.00	0.00	0.00	0.00	0.00	0.00	0.00	0.00	0.00	0.00	0.02	0.00	0.00	0.00	0.01	0.01
na	0.14	0.17	0.13	0.14	0.17	0.22	0.19	0.21	0.18	0.20	0.30	0.22	0.35	0.32	0.44	0.41

* Unclassified order
