# Supplementary material for: Effect of Methane Inhibitors on Ruminal Microbiota During Early Life and Its Relationship With Ruminal Metabolism and Growth in Calves
Source: Front Microbiol. 2021 Sep 16;12:710914. doi: 10.3389/fmicb.2021.710914 (PMC8482044; doi:10.3389/fmicb.2021.710914)
Supplement: Supplementary file 6 [file Table_5.pdf]

Supplementary Table 5. Family composition of the ruminal bacteria in control (Ctrl) and treated (Trt) calved across the different sampling times (weeks) of rearing. Bacterial families highlighted in bold are the most abundant in the rumen of calves.

Time (weeks)	2		4		6		8		10		14		24		49	
**Treatment**	**Ctrl**	**Trt**	**Ctrl**	**Trt**	**Ctrl**	**Trt**	**Ctrl**	**Trt**	**Ctrl**	**Trt**	**Ctrl**	**Trt**	**Ctrl**	**Trt**	**Ctrl**	**Trt**
**Prevotellaceae**	32.71	31.69	26.45	36.22	23.73	17.87	23.41	16.15	23.79	27.48	25.39	23.57	32.83	34.02	46.78	47.21
**Ruminococcaceae**	12.72	10.11	9.34	6.50	19.44	16.51	18.27	16.19	18.27	15.65	25.77	15.83	19.34	19.28	10.46	10.39
**Lachnospiraceae**	18.21	14.89	18.73	13.90	11.39	17.46	8.05	14.12	7.91	8.34	10.20	11.42	16.63	14.72	10.06	9.86
**Succinivibrionaceae**	9.38	14.75	12.66	15.47	4.58	7.97	11.37	5.83	7.93	3.75	3.82	4.30	0.11	0.07	0.16	0.17
**Christensenellaceae**	1.97	0.92	2.62	1.27	6.08	6.37	5.41	10.25	6.85	7.49	5.43	9.07	4.20	4.84	5.58	5.43
**Erysipelotrichaceae**	9.07	10.34	5.30	4.43	3.11	2.77	3.65	5.03	11.85	5.47	3.31	4.22	6.38	5.09	1.00	0.78
**Rikenellaceae**	1.44	3.54	2.47	3.12	3.81	6.24	3.89	8.41	3.61	6.72	4.47	9.99	2.20	2.56	4.98	4.73
**Bacteroidales BS11 gut group**	2.70	0.56	9.45	2.62	4.53	2.12	3.28	2.83	3.16	2.38	4.35	2.33	1.83	2.06	2.92	3.20
**Bacteroidales S24-7 group**	0.59	0.84	3.48	2.34	6.06	3.75	5.33	3.39	3.38	3.25	1.86	2.89	2.23	2.34	2.03	2.54
**Spirochaetaceae**	0.68	1.24	1.35	2.47	7.81	3.32	6.11	3.04	2.12	3.39	0.89	1.87	0.99	1.07	0.95	1.10
**Acidaminococcaceae**	1.73	2.56	1.62	3.18	1.18	2.52	1.67	2.15	0.92	2.24	0.76	1.24	1.03	1.23	1.71	1.52
**p-2534-18B5 gut group**	0.02	0.08	0.03	1.25	0.09	7.38	3.26	5.51	2.09	3.00	0.67	3.23	0.22	0.32	0.02	0.03
**Veillonellaceae**	3.64	1.63	1.57	1.47	0.74	0.37	0.51	0.52	0.59	0.74	0.62	0.71	4.28	3.99	1.43	1.28
**Fibrobacteraceae**	0.04	0.15	0.12	0.27	1.19	0.12	0.83	0.84	1.72	2.33	0.91	0.80	0.55	0.91	1.23	1.92
**Coriobacteriaceae**	1.13	0.95	1.17	1.61	1.00	0.47	0.75	0.74	0.69	1.77	0.63	0.62	0.48	0.54	0.45	0.32
**Mollicutes RF9 ***	0.08	0.16	0.30	0.60	0.39	0.72	0.57	1.02	0.78	0.87	1.50	1.93	1.16	1.21	0.91	0.87
**Family XIII**	0.14	0.11	0.25	0.25	0.42	0.55	0.44	0.59	0.50	0.51	0.62	0.55	1.21	1.31	1.04	0.92
**Bacteroidales RF16 group**	0.02	0.03	0.00	0.13	0.16	0.25	0.36	0.37	0.57	0.20	1.40	0.15	0.65	0.77	1.55	1.47
**Bacteroidaceae**	1.75	1.84	0.29	0.59	0.41	0.17	0.18	0.12	0.15	0.17	0.39	0.21	0.13	0.14	0.31	0.33
**Rhodospirillaceae**	0.04	0.00	0.04	0.09	0.27	0.15	0.31	0.34	0.41	0.24	1.06	0.81	0.37	0.25	1.01	0.74
**Streptococcaceae**	0.02	0.03	0.01	0.02	0.03	0.12	0.03	0.08	0.22	2.04	0.37	1.67	0.35	0.69	0.12	0.09
**Gastranaerophilales ***	0.03	0.01	0.02	0.01	0.14	0.11	0.23	0.06	0.40	0.07	0.82	0.13	0.36	0.30	0.98	1.00

Bacteroidales UCG-001	0.01	0.02	0.04	0.24	0.36	0.32	0.45	0.47	0.17	0.54	0.18	0.95	0.18	0.13	0.20	0.16
SHA 109 *	0.01	0.01	0.23	0.03	0.98	1.16	0.17	0.43	0.58	0.14	0.13	0.05	0.19	0.09	0.05	0.05
Desulfovibrionaceae	0.31	1.97	0.20	1.08	0.05	0.10	0.03	0.04	0.01	0.01	0.01	0.01	0.01	0.02	0.03	0.03
Clostridiales vadinBB60 group	0.15	0.10	0.95	0.10	0.49	0.21	0.18	0.41	0.16	0.24	0.37	0.25	0.05	0.03	0.08	0.09
Clostridiaceae 1	0.40	0.36	0.02	0.00	0.11	0.01	0.00	0.00	0.00	0.00	1.65	0.08	0.00	0.00	0.01	0.01
Saccharibacteria *	0.00	0.00	0.00	0.00	0.09	0.02	0.06	0.09	0.14	0.11	0.11	0.20	0.32	0.44	0.49	0.41
Anaeroplasmataceae	0.05	0.00	0.01	0.01	0.08	0.04	0.04	0.02	0.01	0.03	0.18	0.04	0.28	0.32	0.39	0.51
Porphyromonadaceae	0.26	0.29	0.06	0.11	0.03	0.04	0.04	0.15	0.05	0.05	0.12	0.08	0.04	0.07	0.09	0.06
Enterobacteriaceae	0.01	0.00	0.00	0.00	0.00	0.01	0.01	0.01	0.01	0.01	0.00	0.01	0.02	0.01	0.64	0.67
Victivallaceae	0.00	0.00	0.00	0.00	0.06	0.00	0.09	0.00	0.09	0.00	0.40	0.00	0.05	0.06	0.13	0.12
Bacteroidales Incertae Sedis	0.06	0.23	0.19	0.13	0.08	0.06	0.06	0.04	0.03	0.02	0.01	0.03	0.03	0.02	0.00	0.00
Anaerolineaceae	0.01	0.00	0.05	0.02	0.14	0.03	0.09	0.05	0.07	0.05	0.06	0.03	0.04	0.04	0.06	0.06
Lentisphaerae RFP12 gut group *	0.02	0.00	0.04	0.01	0.06	0.02	0.06	0.05	0.05	0.03	0.12	0.05	0.03	0.03	0.09	0.09
RH-aaj90h05	0.04	0.01	0.19	0.05	0.10	0.05	0.07	0.06	0.05	0.02	0.02	0.01	0.02	0.01	0.01	0.00
GR-WP33-58	0.01	0.00	0.03	0.00	0.06	0.08	0.06	0.03	0.02	0.06	0.03	0.06	0.06	0.05	0.06	0.09
Alcaligenaceae	0.01	0.02	0.01	0.02	0.01	0.05	0.01	0.02	0.03	0.06	0.02	0.02	0.03	0.05	0.15	0.17
Lineage I (Endomicrobia) *	0.01	0.00	0.28	0.00	0.11	0.00	0.05	0.00	0.06	0.00	0.11	0.00	0.00	0.00	0.01	0.01
Synergistaceae	0.02	0.06	0.03	0.05	0.07	0.01	0.07	0.04	0.05	0.01	0.09	0.01	0.02	0.01	0.04	0.04
Candidate division SR1 *	0.00	0.00	0.00	0.00	0.00	0.00	0.00	0.00	0.00	0.00	0.00	0.03	0.10	0.06	0.20	0.18
Defluviitaleaceae	0.01	0.00	0.00	0.00	0.00	0.01	0.05	0.04	0.02	0.07	0.01	0.02	0.05	0.06	0.07	0.08
Lactobacillaceae	0.05	0.04	0.03	0.02	0.03	0.03	0.01	0.02	0.05	0.04	0.02	0.04	0.07	0.01	0.00	0.00
Marinilabiaceae	0.00	0.00	0.00	0.00	0.00	0.00	0.00	0.00	0.01	0.00	0.38	0.00	0.02	0.01	0.02	0.02
NB1-n *	0.06	0.03	0.09	0.02	0.08	0.00	0.01	0.01	0.01	0.00	0.02	0.01	0.02	0.01	0.05	0.04
Bacteroidetes VC2.1 Bac22 *	0.00	0.00	0.00	0.00	0.00	0.00	0.02	0.00	0.01	0.00	0.01	0.00	0.05	0.07	0.11	0.06
Planctomycetaceae	0.00	0.00	0.01	0.00	0.04	0.03	0.04	0.02	0.02	0.01	0.03	0.02	0.01	0.02	0.04	0.02
Campylobacteraceae	0.07	0.05	0.03	0.04	0.02	0.01	0.03	0.01	0.02	0.01	0.01	0.00	0.00	0.01	0.01	0.00
Elusimicrobiaceae	0.04	0.01	0.00	0.00	0.05	0.01	0.02	0.00	0.03	0.00	0.03	0.01	0.02	0.01	0.03	0.04
Neisseriaceae	0.06	0.04	0.02	0.03	0.01	0.02	0.02	0.01	0.01	0.02	0.00	0.01	0.01	0.01	0.01	0.01
Bacteroidales *	0.01	0.06	0.01	0.01	0.02	0.01	0.01	0.03	0.01	0.00	0.03	0.01	0.02	0.02	0.02	0.02

Rhizobiaceae	0.00	0.00	0.00	0.00	0.00	0.00	0.01	0.01	0.01	0.01	0.01	0.02	0.01	0.01	0.09	0.08
Oligosphaeraceae	0.00	0.00	0.00	0.00	0.00	0.00	0.00	0.00	0.00	0.00	0.04	0.00	0.02	0.03	0.07	0.05
Bacteroidetes BD2-2 *	0.00	0.00	0.00	0.00	0.00	0.00	0.00	0.00	0.00	0.01	0.07	0.01	0.06	0.03	0.01	0.02
Pseudomonadaceae	0.00	0.00	0.00	0.00	0.00	0.01	0.00	0.01	0.01	0.01	0.00	0.01	0.05	0.02	0.06	0.04
Eubacteriaceae	0.02	0.03	0.03	0.03	0.03	0.02	0.02	0.02	0.01	0.01	0.00	0.00	0.00	0.00	0.00	0.00
Chloroplast *	0.00	0.00	0.00	0.00	0.01	0.01	0.01	0.01	0.01	0.01	0.02	0.01	0.06	0.04	0.01	0.01
Pasteurellaceae	0.01	0.01	0.01	0.01	0.01	0.01	0.01	0.01	0.02	0.02	0.01	0.01	0.02	0.02	0.03	0.02
Methylobacteriaceae	0.00	0.00	0.00	0.00	0.00	0.00	0.00	0.00	0.00	0.01	0.01	0.01	0.01	0.01	0.06	0.07
Sphingomonadaceae	0.00	0.00	0.00	0.00	0.00	0.00	0.00	0.00	0.00	0.02	0.02	0.02	0.03	0.03	0.02	0.02
Peptococcaceae	0.00	0.00	0.00	0.00	0.01	0.00	0.02	0.00	0.01	0.00	0.03	0.00	0.01	0.01	0.02	0.02
Oligoflexales *	0.00	0.00	0.00	0.00	0.00	0.00	0.00	0.00	0.00	0.00	0.00	0.00	0.00	0.00	0.10	0.04
Corynebacteriaceae	0.01	0.01	0.00	0.01	0.00	0.01	0.01	0.02	0.01	0.02	0.01	0.01	0.01	0.01	0.00	0.00
Nocardiaceae	0.00	0.00	0.00	0.00	0.01	0.01	0.00	0.00	0.00	0.00	0.00	0.01	0.02	0.01	0.04	0.04
Rickettsiales Incertae Sedis	0.00	0.01	0.00	0.00	0.00	0.00	0.00	0.00	0.00	0.01	0.01	0.02	0.00	0.00	0.05	0.04
Microbacteriaceae	0.00	0.00	0.00	0.00	0.00	0.01	0.01	0.01	0.00	0.01	0.01	0.02	0.01	0.01	0.02	0.02
Moraxellaceae	0.01	0.00	0.00	0.01	0.01	0.01	0.01	0.00	0.01	0.01	0.00	0.01	0.01	0.01	0.01	0.01
Acholeplasmataceae	0.03	0.00	0.00	0.00	0.00	0.03	0.00	0.01	0.00	0.00	0.00	0.00	0.00	0.01	0.00	0.00
PL-11B10	0.00	0.00	0.00	0.00	0.00	0.01	0.00	0.02	0.01	0.01	0.00	0.01	0.00	0.00	0.01	0.02
PeH15	0.00	0.00	0.00	0.00	0.00	0.00	0.00	0.00	0.00	0.00	0.00	0.00	0.00	0.00	0.03	0.04
Comamonadaceae	0.01	0.02	0.00	0.00	0.01	0.01	0.00	0.00	0.00	0.00	0.00	0.00	0.00	0.00	0.00	0.00
Armatimonadetes *	0.00	0.00	0.00	0.00	0.00	0.00	0.00	0.00	0.00	0.00	0.01	0.00	0.01	0.01	0.02	0.02
UCT N117 *	0.00	0.00	0.00	0.00	0.01	0.00	0.01	0.00	0.01	0.00	0.01	0.00	0.00	0.00	0.01	0.02
Flavobacteriaceae	0.00	0.00	0.00	0.00	0.00	0.01	0.00	0.00	0.01	0.01	0.00	0.00	0.01	0.01	0.00	0.00
Caulobacteraceae	0.00	0.00	0.00	0.00	0.01	0.01	0.00	0.00	0.01	0.01	0.00	0.01	0.00	0.00	0.00	0.00
MSBL5 *	0.00	0.00	0.00	0.00	0.00	0.00	0.00	0.00	0.00	0.00	0.00	0.00	0.02	0.00	0.02	0.01
WCHB1-25 *	0.00	0.00	0.00	0.01	0.00	0.00	0.00	0.00	0.00	0.00	0.00	0.00	0.00	0.00	0.01	0.00
Desulfobulbaceae	0.00	0.00	0.00	0.00	0.00	0.01	0.00	0.00	0.00	0.01	0.00	0.00	0.00	0.00	0.00	0.00
Family Incertae Sedis	0.00	0.00	0.00	0.00	0.00	0.00	0.00	0.01	0.01	0.01	0.01	0.01	0.00	0.00	0.00	0.00
Bacillaceae	0.00	0.00	0.00	0.00	0.00	0.00	0.00	0.00	0.00	0.00	0.00	0.00	0.00	0.00	0.01	0.02

WA-aaa01f12	0.00	0.00	0.00	0.00	0.00	0.00	0.00	0.00	0.00	0.00	0.02	0.00	0.00	0.00	0.01	0.01
na	0.14	0.17	0.13	0.14	0.17	0.22	0.19	0.21	0.18	0.20	0.30	0.22	0.35	0.32	0.44	0.41

* Unclassified family
