# Supplementary material for: Effect of Methane Inhibitors on Ruminal Microbiota During Early Life and Its Relationship With Ruminal Metabolism and Growth in Calves
Source: Front Microbiol. 2021 Sep 16;12:710914. doi: 10.3389/fmicb.2021.710914 (PMC8482044; doi:10.3389/fmicb.2021.710914)
Supplement: Supplementary file 7 [file Table_6.pdf]

Supplementary Table 6. Genus composition of the ruminal bacteria in control (Ctrl) and treated (Trt) calved across the different sampling times (weeks) of rearing. Bacterial genera highlighted in bold are the most abundant in the rumen of calves.

**Time (weeks)**	**2**		**4**		**6**		**8**		**10**		**14**		**24**		**49**	
**Treatment**	**Ctrl**	**Trt**	**Ctrl**	**Trt**	**Ctrl**	**Trt**	**Ctrl**	**Trt**	**Ctrl**	**Trt**	**Ctrl**	**Trt**	**Ctrl**	**Trt**	**Ctrl**	**Trt**
**Prevotella 1**	24.11	25.41	21.49	29.31	21.30	15.09	20.88	13.83	19.24	25.01	20.91	21.17	28.64	29.72	40.01	40.22
**Christensenellaceae R-7 group**	1.96	0.91	2.60	1.26	6.07	6.36	5.39	10.25	6.81	7.49	5.40	9.07	4.19	4.83	5.57	5.42
**Rikenellaceae RC9 gut group**	1.02	3.41	2.21	2.98	3.38	6.13	3.71	8.29	3.53	6.64	4.44	9.88	2.16	2.51	4.91	4.59
**Ruminococcus 2**	5.98	5.24	2.24	1.66	7.14	8.15	6.66	4.12	8.03	4.97	6.56	3.22	0.48	0.59	0.31	0.27
**Sharpea**	8.85	10.08	2.45	4.20	2.04	2.34	3.13	4.65	7.69	4.61	0.44	0.99	0.01	0.01	0.00	0.01
**Bacteroidales BS11 gut group ***	2.70	0.56	9.45	2.62	4.53	2.12	3.28	2.83	3.16	2.38	4.35	2.33	1.83	2.06	2.92	3.20
**Bacteroidales S24-7 group ***	0.59	0.84	3.48	2.34	6.06	3.75	5.33	3.39	3.38	3.25	1.86	2.89	2.23	2.34	2.03	2.54
**Ruminobacter**	0.02	0.12	4.45	2.95	0.13	7.20	7.95	3.14	7.03	2.96	3.38	3.96	0.03	0.02	0.03	0.03
**Ruminiclostridium 9**	0.05	0.10	0.09	0.13	0.76	1.39	1.35	2.95	1.68	3.55	7.47	5.08	6.49	7.67	0.40	0.37
**Lachnospiraceae UCG-005**	10.25	4.99	8.43	0.43	0.03	3.19	0.02	2.84	0.01	0.03	0.01	0.01	0.02	0.01	0.05	0.03
**Treponema 2**	0.11	0.94	0.98	2.03	7.23	2.92	4.37	2.23	1.48	1.93	0.53	1.01	0.87	0.93	0.80	0.95
**Roseburia**	3.34	5.53	1.77	7.28	0.90	4.79	0.08	0.31	0.10	0.17	0.35	0.18	1.74	1.28	0.53	0.42
**p-2534-18B5 gut group ***	0.02	0.08	0.03	1.25	0.09	7.38	3.26	5.51	2.09	3.00	0.67	3.23	0.22	0.32	0.02	0.03
**Lachnospiraceae NK3A20 group**	0.02	0.10	0.15	1.06	0.32	4.04	0.81	2.27	2.02	2.28	3.65	5.33	1.27	1.28	1.24	1.18
**Succiniclasticum**	0.95	1.90	1.49	2.89	1.17	2.51	1.66	2.13	0.92	2.23	0.75	1.24	1.03	1.23	1.71	1.52
**Succinivibrio**	3.06	9.48	5.54	3.82	1.69	0.14	0.28	0.03	0.70	0.32	0.10	0.06	0.00	0.00	0.02	0.02
**Ruminococcus 1**	0.46	0.37	0.71	0.59	1.43	0.80	1.61	1.29	2.06	1.64	1.99	1.58	3.37	2.55	1.79	1.80
**Ruminococcaceae UCG-014**	1.42	1.55	0.74	1.74	1.20	2.69	1.55	1.82	2.01	1.16	2.26	1.09	1.28	1.29	1.03	0.96
**Succinivibrionaceae UCG-002**	3.09	0.11	1.68	7.97	2.68	0.41	2.86	2.55	0.15	0.32	0.32	0.21	0.07	0.03	0.08	0.09
**Ruminococcaceae NK4A214 group**	1.49	0.17	0.44	0.37	0.93	0.74	0.99	0.74	0.96	0.62	0.91	0.71	2.89	2.64	2.46	2.80
**Ruminiclostridium 5**	1.77	0.91	2.55	0.15	3.68	0.19	2.32	1.76	0.66	0.71	1.94	0.29	0.31	0.34	0.25	0.25
**Prevotellaceae UCG-001**	1.32	0.95	1.53	1.32	1.03	0.43	0.60	0.43	0.77	0.48	1.55	0.40	1.00	1.28	1.83	2.27

**Prevotellaceae UCG-003**	0.25	0.32	0.41	0.57	0.63	0.49	0.82	0.59	2.16	0.61	1.67	0.66	0.93	1.43	2.84	2.53
**Selenomonas 1**	0.87	0.69	0.32	0.60	0.27	0.19	0.19	0.40	0.28	0.66	0.38	0.60	3.74	3.36	0.97	0.90
**Fibrobacter**	0.04	0.15	0.12	0.27	1.19	0.12	0.83	0.84	1.72	2.33	0.91	0.80	0.55	0.91	1.23	1.92
**Pseudobutyrivibrio**	0.51	0.45	0.64	0.37	0.32	0.21	0.25	0.45	0.23	0.35	1.01	0.69	2.02	2.54	1.52	1.52
**Mollicutes RF9 ***	0.08	0.16	0.30	0.60	0.39	0.72	0.57	1.02	0.78	0.87	1.50	1.93	1.16	1.21	0.91	0.87
**Prevotella 7**	6.08	2.51	1.87	0.85	0.05	0.08	0.02	0.03	0.07	0.02	0.01	0.01	0.50	0.30	0.04	0.03
**Kandleria**	0.00	0.00	0.00	0.00	0.01	0.00	0.05	0.04	1.34	0.38	0.10	0.16	5.56	4.33	0.29	0.05
**Succinimonas**	3.19	5.02	0.96	0.71	0.06	0.22	0.26	0.10	0.04	0.13	0.00	0.04	0.00	0.00	0.01	0.01
**Ruminococcaceae UCG-005**	0.22	0.07	0.94	0.15	0.88	0.29	0.96	1.21	0.60	0.99	0.93	0.65	0.64	0.68	0.63	0.49
**Eubacterium coprostanoligenes group**	0.06	0.05	0.21	0.19	0.74	0.33	0.79	0.45	0.77	0.39	1.14	0.58	1.04	1.10	1.02	0.95
**Erysipelotrichaceae UCG-002**	0.00	0.00	1.11	0.00	0.54	0.00	0.20	0.02	2.40	0.03	2.33	2.66	0.26	0.11	0.00	0.00
**Atopobium**	0.61	0.24	0.56	0.94	0.87	0.15	0.67	0.60	0.62	1.69	0.58	0.55	0.32	0.35	0.33	0.24
**Lachnospiraceae ****	0.25	0.28	0.34	0.20	3.81	0.51	1.20	0.58	0.22	0.26	0.20	0.36	0.23	0.23	0.27	0.26
**Prevotellaceae NK3B31 group**	0.02	1.46	0.31	2.47	0.29	0.36	0.50	0.20	0.90	0.14	0.72	0.08	0.54	0.31	0.31	0.29
**Sphaerochaeta**	0.57	0.30	0.37	0.45	0.58	0.40	1.74	0.81	0.63	1.47	0.34	0.86	0.11	0.13	0.06	0.03
**Lachnospiraceae NK4A136 group**	0.19	0.45	0.73	0.32	0.50	0.64	0.46	0.46	0.43	0.40	0.70	0.98	0.71	0.61	0.46	0.41
**Ruminococcaceae UCG-002**	0.22	0.83	0.37	0.73	0.50	0.78	0.30	0.75	0.30	0.60	0.39	0.91	0.53	0.42	0.38	0.38
**Bacteroidales RF16 group ***	0.02	0.03	0.00	0.13	0.16	0.25	0.36	0.37	0.57	0.20	1.40	0.15	0.65	0.77	1.55	1.47
Alloprevotella	0.64	0.83	0.65	1.26	0.34	1.19	0.40	0.70	0.24	0.64	0.13	0.42	0.11	0.13	0.15	0.11
**Eubacterium ventriosum group**	0.95	0.06	3.58	2.40	0.27	0.02	0.09	0.10	0.09	0.03	0.06	0.05	0.04	0.04	0.06	0.05
Saccharofermentans	0.02	0.00	0.10	0.01	0.41	0.37	0.99	0.41	0.57	0.36	1.11	0.33	1.20	1.03	0.49	0.47
Eubacterium oxidoreducens group	0.00	0.00	0.01	0.00	0.50	0.04	1.75	2.18	1.23	1.25	0.41	0.17	0.01	0.01	0.03	0.03
Bacteroides	1.75	1.84	0.29	0.59	0.41	0.17	0.18	0.12	0.15	0.17	0.39	0.21	0.13	0.14	0.31	0.33
Eubacterium ruminantium group	0.12	0.06	0.07	0.31	0.13	0.16	0.51	0.28	0.35	0.15	0.28	0.32	1.53	0.94	0.34	0.36
Streptococcus	0.02	0.03	0.01	0.02	0.03	0.12	0.03	0.08	0.22	2.04	0.37	1.67	0.35	0.69	0.12	0.09
Eubacterium hallii group	0.06	0.33	0.13	0.15	0.40	0.55	0.32	1.02	0.46	0.61	0.23	0.38	0.33	0.36	0.24	0.22
Thalassospira	0.03	0.00	0.03	0.09	0.25	0.14	0.27	0.34	0.39	0.23	0.97	0.77	0.35	0.24	0.96	0.69

Butyrivibrio 2	0.01	0.01	0.02	0.00	0.04	0.01	0.04	0.12	0.07	0.18	0.27	0.20	1.21	1.14	1.24	1.18
Ruminococcus gauvreauii group	0.40	0.18	0.88	0.13	0.80	0.26	0.24	0.19	0.77	0.13	0.36	0.06	0.34	0.36	0.17	0.17
Gastranaerophilales *	0.03	0.01	0.02	0.01	0.14	0.11	0.23	0.06	0.40	0.07	0.82	0.13	0.36	0.30	0.98	1.00
Bacteroidales UCG-001 *	0.01	0.02	0.04	0.24	0.36	0.32	0.45	0.47	0.17	0.54	0.18	0.95	0.18	0.13	0.20	0.16
Acetitomaculum	0.02	0.00	0.19	0.12	1.04	0.35	0.48	0.54	0.19	0.27	0.21	0.22	0.15	0.17	0.22	0.20
Blautia	0.70	0.98	0.31	0.25	0.22	0.31	0.12	0.26	0.14	0.40	0.07	0.08	0.10	0.10	0.15	0.14
Ruminococcaceae UCG-010	0.04	0.01	0.03	0.03	0.28	0.10	0.15	0.14	0.14	0.19	0.26	1.05	0.28	0.33	0.66	0.63
SHA 109 *	0.01	0.01	0.23	0.03	0.98	1.16	0.17	0.43	0.58	0.14	0.13	0.05	0.19	0.09	0.05	0.05
Erysipelotrichaceae UCG-004	0.18	0.21	1.48	0.08	0.25	0.20	0.08	0.08	0.16	0.10	0.14	0.05	0.18	0.15	0.28	0.30
Desulfovibrio	0.31	1.97	0.20	1.08	0.05	0.10	0.03	0.04	0.01	0.01	0.01	0.01	0.01	0.02	0.03	0.03
Christensenellaceae *	0.15	0.10	0.95	0.10	0.49	0.21	0.18	0.41	0.16	0.24	0.37	0.25	0.05	0.03	0.08	0.09
Lachnospiraceae AC2044 group	0.00	0.00	0.05	0.00	0.04	0.01	0.04	0.08	0.05	0.04	0.20	0.57	0.46	1.07	0.52	0.54
Prevotellaceae **	0.02	0.08	0.00	0.09	0.00	0.15	0.01	0.14	0.01	0.15	0.04	0.53	0.18	0.10	0.96	1.05
Mogibacterium	0.06	0.06	0.10	0.17	0.14	0.30	0.11	0.27	0.11	0.20	0.14	0.17	0.46	0.51	0.36	0.31
Coprococcus 2	0.10	0.14	0.15	0.03	0.06	0.05	0.24	0.28	0.24	0.33	0.33	0.25	0.65	0.23	0.05	0.04
Olsenella	0.48	0.69	0.57	0.61	0.08	0.26	0.05	0.06	0.04	0.02	0.00	0.00	0.01	0.01	0.01	0.01
Lachnospiraceae ND3007 group	0.00	0.00	0.03	0.01	0.00	0.02	0.01	0.01	0.01	0.01	0.01	0.05	1.19	0.76	0.34	0.41
Oribacterium	0.09	0.03	0.04	0.07	0.13	0.04	0.06	0.03	0.05	0.03	0.06	0.03	0.41	0.43	0.63	0.67
Lachnospiraceae XPB1014 group	0.00	0.00	0.00	0.00	0.01	0.00	0.02	0.04	0.02	0.03	0.09	0.02	1.32	0.47	0.36	0.37
Clostridium sensu stricto 1	0.40	0.36	0.02	0.00	0.11	0.01	0.00	0.00	0.00	0.00	1.65	0.08	0.00	0.00	0.01	0.01
Syntrophococcus	0.15	0.23	0.40	0.25	0.23	0.18	0.11	0.09	0.15	0.19	0.10	0.11	0.05	0.11	0.07	0.07
Candidatus Saccharimonas	0.00	0.00	0.00	0.00	0.09	0.02	0.06	0.09	0.14	0.11	0.11	0.20	0.32	0.44	0.49	0.41
Anaerosporobacter	0.02	0.00	0.14	0.01	0.68	0.15	0.18	1.04	0.07	0.04	0.06	0.02	0.00	0.02	0.00	0.00
Lachnoclostridium	0.68	0.69	0.23	0.22	0.07	0.16	0.03	0.06	0.02	0.03	0.02	0.03	0.05	0.04	0.03	0.02
Anaerotruncus	0.05	0.05	0.46	0.09	0.88	0.09	0.10	0.06	0.05	0.05	0.12	0.04	0.08	0.09	0.08	0.07
Anaerovibrio	0.36	0.10	0.48	0.23	0.16	0.04	0.11	0.02	0.06	0.03	0.06	0.03	0.08	0.12	0.20	0.16
Family XIII AD3011 group	0.03	0.00	0.07	0.01	0.14	0.06	0.15	0.06	0.16	0.07	0.26	0.08	0.31	0.32	0.25	0.21

Anaeroplasma	0.05	0.00	0.01	0.01	0.08	0.04	0.04	0.02	0.01	0.03	0.18	0.04	0.28	0.32	0.39	0.51
Incertae Sedis	0.01	0.01	0.02	0.01	0.05	0.03	0.02	0.05	0.03	0.04	0.36	0.03	0.62	0.40	0.13	0.10
Prevotellaceae YAB2003 group	0.04	0.03	0.02	0.04	0.01	0.01	0.01	0.01	0.01	0.03	0.13	0.06	0.70	0.43	0.16	0.19
Shuttleworthia	0.01	0.07	0.01	0.02	0.00	0.86	0.07	0.02	0.02	0.01	0.09	0.02	0.36	0.22	0.05	0.05
Marvinbryantia	0.01	0.00	0.01	0.02	0.17	0.25	0.15	0.07	0.18	0.18	0.13	0.13	0.10	0.09	0.13	0.15
Ruminiclostridium 6	0.02	0.02	0.05	0.06	0.16	0.03	0.15	0.07	0.11	0.07	0.16	0.07	0.20	0.13	0.22	0.24
Coprococcus 1	0.07	0.04	0.05	0.04	0.07	0.05	0.20	0.13	0.21	0.05	0.08	0.01	0.22	0.26	0.11	0.11
Eubacterium nodatum group	0.01	0.01	0.01	0.03	0.06	0.13	0.09	0.17	0.12	0.16	0.10	0.20	0.17	0.18	0.12	0.09
Megasphaera	0.38	0.13	0.28	0.31	0.12	0.07	0.08	0.00	0.12	0.00	0.12	0.00	0.00	0.00	0.00	0.00
Prevotellaceae Ga6A1 group	0.01	0.00	0.00	0.02	0.00	0.01	0.06	0.05	0.33	0.19	0.15	0.10	0.04	0.13	0.19	0.27
Selenomonas	1.43	0.01	0.07	0.00	0.00	0.00	0.00	0.00	0.00	0.00	0.00	0.00	0.00	0.00	0.00	0.00
Acidaminococcus	0.56	0.51	0.13	0.28	0.01	0.01	0.01	0.01	0.00	0.00	0.00	0.00	0.00	0.00	0.00	0.00
Ruminococcaceae **	0.17	0.27	0.08	0.17	0.09	0.07	0.05	0.07	0.03	0.06	0.04	0.03	0.12	0.08	0.07	0.08
Veillonellaceae UCG-001	0.08	0.23	0.05	0.08	0.09	0.02	0.07	0.07	0.07	0.04	0.04	0.06	0.09	0.09	0.12	0.11
Prevotellaceae UCG-004	0.01	0.00	0.02	0.04	0.07	0.02	0.11	0.10	0.04	0.17	0.05	0.10	0.06	0.08	0.20	0.15
Ruminococcaceae UCG-004	0.09	0.08	0.08	0.07	0.08	0.09	0.05	0.11	0.07	0.08	0.06	0.06	0.07	0.07	0.06	0.06
Schwartzia	0.21	0.28	0.15	0.12	0.00	0.03	0.01	0.01	0.01	0.01	0.01	0.03	0.07	0.18	0.02	0.02
Prevotella 9	0.20	0.06	0.15	0.23	0.01	0.01	0.00	0.01	0.01	0.01	0.00	0.00	0.13	0.10	0.10	0.10
Lachnospiraceae UCG-008	0.02	0.01	0.01	0.01	0.05	0.04	0.07	0.06	0.08	0.08	0.08	0.10	0.10	0.10	0.13	0.14
SP3-e08	0.08	0.01	0.10	0.03	0.34	0.02	0.13	0.08	0.06	0.04	0.01	0.05	0.03	0.02	0.02	0.03
Lachnospiraceae FCS020 group	0.00	0.00	0.02	0.00	0.01	0.01	0.01	0.01	0.02	0.03	0.05	0.34	0.14	0.18	0.09	0.08
Victivallis	0.00	0.00	0.00	0.00	0.06	0.00	0.09	0.00	0.09	0.00	0.40	0.00	0.05	0.06	0.13	0.12
Anaerovorax	0.01	0.00	0.01	0.02	0.02	0.04	0.05	0.06	0.05	0.05	0.06	0.07	0.12	0.13	0.15	0.14
Phocaeicola	0.06	0.23	0.19	0.13	0.08	0.06	0.06	0.04	0.03	0.02	0.01	0.03	0.03	0.02	0.00	0.00
Erysipelotrichaceae UCG-009	0.00	0.00	0.00	0.01	0.05	0.01	0.02	0.04	0.02	0.05	0.04	0.06	0.20	0.26	0.10	0.11
Erysipelotrichaceae **	0.01	0.01	0.12	0.06	0.08	0.12	0.05	0.07	0.03	0.10	0.03	0.05	0.04	0.06	0.06	0.07
Ruminiclostridium	0.14	0.20	0.12	0.11	0.02	0.11	0.02	0.01	0.02	0.00	0.03	0.00	0.01	0.01	0.06	0.08

Pantoea	0.00	0.00	0.00	0.00	0.00	0.00	0.00	0.00	0.01	0.01	0.00	0.00	0.00	0.00	0.44	0.44
Lachnospiraceae FE2018 group	0.01	0.02	0.02	0.01	0.13	0.11	0.07	0.12	0.03	0.12	0.12	0.14	0.00	0.00	0.01	0.01
Eubacterium cellulosolvens group	0.00	0.00	0.00	0.00	0.00	0.00	0.00	0.00	0.00	0.00	0.00	0.00	0.39	0.43	0.05	0.03
Lachnospiraceae UCG-001	0.00	0.01	0.04	0.01	0.05	0.03	0.12	0.13	0.08	0.14	0.10	0.07	0.02	0.02	0.03	0.02
Mitsuokella	0.27	0.15	0.17	0.09	0.05	0.01	0.03	0.01	0.06	0.00	0.00	0.00	0.00	0.00	0.00	0.00
Ruminococcaceae UCG-013	0.17	0.06	0.02	0.02	0.02	0.04	0.03	0.03	0.04	0.03	0.07	0.06	0.06	0.04	0.08	0.09
probable genus 10	0.00	0.00	0.00	0.00	0.00	0.00	0.00	0.00	0.00	0.00	0.08	0.11	0.11	0.23	0.14	0.15
Solobacterium	0.00	0.00	0.00	0.00	0.00	0.00	0.00	0.03	0.01	0.04	0.16	0.07	0.08	0.09	0.16	0.14
Anaerolineaceae **	0.01	0.00	0.05	0.02	0.14	0.03	0.09	0.05	0.07	0.05	0.06	0.03	0.04	0.04	0.06	0.06
Lentisphaerae RFP12 gut group *	0.02	0.00	0.04	0.01	0.06	0.02	0.06	0.05	0.05	0.03	0.12	0.05	0.03	0.03	0.09	0.09
Moryella	0.01	0.05	0.04	0.02	0.08	0.12	0.07	0.05	0.06	0.07	0.05	0.02	0.02	0.03	0.04	0.02
RH-aaj90h05 *	0.04	0.01	0.19	0.05	0.10	0.05	0.07	0.06	0.05	0.02	0.02	0.01	0.02	0.01	0.01	0.00
GR-WP33-58 *	0.01	0.00	0.03	0.00	0.06	0.08	0.06	0.03	0.02	0.06	0.03	0.06	0.06	0.05	0.06	0.09
Tyzzerella 3	0.00	0.00	0.00	0.00	0.00	0.00	0.01	0.00	0.01	0.00	0.04	0.00	0.11	0.13	0.17	0.19
Sutterella	0.01	0.02	0.01	0.02	0.01	0.05	0.01	0.02	0.03	0.06	0.02	0.02	0.03	0.05	0.15	0.17
U29-B03	0.07	0.01	0.15	0.08	0.06	0.07	0.01	0.02	0.01	0.02	0.01	0.02	0.01	0.02	0.04	0.09
Lineage I (Endomicrobia) *	0.01	0.00	0.28	0.00	0.11	0.00	0.05	0.00	0.06	0.00	0.11	0.00	0.00	0.00	0.01	0.01
Anaerorhabdus furcosa group	0.01	0.00	0.06	0.01	0.05	0.03	0.05	0.04	0.07	0.03	0.02	0.03	0.05	0.05	0.07	0.08
Howardella	0.07	0.06	0.05	0.05	0.04	0.04	0.04	0.06	0.03	0.08	0.02	0.05	0.01	0.01	0.01	0.02
Quinella	0.00	0.00	0.00	0.00	0.00	0.00	0.00	0.00	0.00	0.00	0.00	0.00	0.27	0.20	0.08	0.06
Papillibacter	0.00	0.00	0.01	0.00	0.02	0.00	0.07	0.02	0.08	0.01	0.10	0.02	0.02	0.02	0.13	0.11
Candidate division SR1 *	0.00	0.00	0.00	0.00	0.00	0.00	0.00	0.00	0.00	0.00	0.00	0.03	0.10	0.06	0.20	0.18
Erysipelotrichaceae UCG-001	0.01	0.00	0.05	0.03	0.06	0.04	0.03	0.04	0.07	0.10	0.02	0.10	0.00	0.00	0.00	0.00
Lachnospiraceae UCG-006	0.01	0.00	0.01	0.00	0.02	0.01	0.06	0.01	0.05	0.03	0.04	0.02	0.03	0.05	0.09	0.09
Family XIII UCG-001	0.01	0.02	0.01	0.01	0.01	0.02	0.01	0.01	0.01	0.02	0.02	0.02	0.08	0.09	0.08	0.08
Ruminococcaceae UCG-007	0.00	0.00	0.00	0.00	0.00	0.00	0.01	0.00	0.01	0.00	0.01	0.01	0.13	0.13	0.13	0.07
Defluviitaleaceae UCG-011	0.01	0.00	0.00	0.00	0.00	0.01	0.05	0.04	0.02	0.07	0.01	0.02	0.05	0.06	0.07	0.08

Lactobacillus	0.05	0.04	0.03	0.02	0.03	0.03	0.01	0.02	0.05	0.04	0.02	0.04	0.07	0.01	0.00	0.00
Marinilabiaceae **	0.00	0.00	0.00	0.00	0.00	0.00	0.00	0.00	0.01	0.00	0.38	0.00	0.02	0.01	0.02	0.02
Alistipes	0.27	0.11	0.02	0.02	0.01	0.01	0.01	0.01	0.00	0.00	0.00	0.01	0.00	0.00	0.00	0.00
Ruminococcaceae UCG-003	0.02	0.04	0.01	0.13	0.01	0.16	0.01	0.03	0.00	0.00	0.01	0.01	0.00	0.00	0.01	0.01
NB1-n *	0.06	0.03	0.09	0.02	0.08	0.00	0.01	0.01	0.01	0.00	0.02	0.01	0.02	0.01	0.05	0.04
Subdoligranulum	0.25	0.07	0.04	0.04	0.00	0.00	0.00	0.00	0.00	0.00	0.00	0.00	0.00	0.00	0.00	0.00
Lachnospira	0.00	0.00	0.00	0.00	0.01	0.00	0.01	0.01	0.00	0.00	0.00	0.01	0.06	0.06	0.11	0.14
Coriobacteriaceae **	0.03	0.01	0.03	0.02	0.03	0.02	0.02	0.04	0.02	0.01	0.01	0.01	0.04	0.05	0.03	0.03
Phascolarctobacterium	0.22	0.15	0.00	0.00	0.00	0.00	0.00	0.00	0.00	0.00	0.00	0.00	0.00	0.00	0.00	0.00
Lachnospiraceae UCG-002	0.01	0.00	0.01	0.01	0.02	0.02	0.02	0.03	0.03	0.02	0.03	0.03	0.02	0.02	0.05	0.05
Rhodospirillaceae **	0.01	0.00	0.01	0.00	0.02	0.00	0.03	0.00	0.02	0.01	0.09	0.04	0.01	0.01	0.05	0.06
Ruminococcaceae V9D2013 group	0.00	0.00	0.01	0.00	0.14	0.00	0.02	0.00	0.01	0.00	0.06	0.00	0.01	0.01	0.04	0.05
Anaerostipes	0.01	0.00	0.00	0.01	0.02	0.01	0.02	0.04	0.02	0.07	0.02	0.05	0.03	0.02	0.03	0.02
Porphyromonadaceae **	0.00	0.00	0.01	0.01	0.01	0.00	0.03	0.01	0.02	0.00	0.05	0.04	0.01	0.04	0.06	0.05
Butyricimonas	0.12	0.13	0.02	0.06	0.01	0.02	0.00	0.00	0.00	0.00	0.00	0.00	0.00	0.00	0.00	0.00
Dorea	0.05	0.04	0.05	0.03	0.02	0.10	0.01	0.01	0.01	0.00	0.00	0.00	0.00	0.00	0.00	0.00
Parabacteroides	0.12	0.13	0.01	0.02	0.01	0.00	0.00	0.00	0.00	0.00	0.03	0.00	0.00	0.00	0.00	0.00
Bacteroidetes VC2.1 Bac22 *	0.00	0.00	0.00	0.00	0.00	0.00	0.02	0.00	0.01	0.00	0.01	0.00	0.05	0.07	0.11	0.06
Proteiniphilum	0.00	0.00	0.00	0.00	0.00	0.02	0.01	0.13	0.02	0.04	0.02	0.03	0.03	0.02	0.02	0.01
Pyramidobacter	0.01	0.04	0.02	0.04	0.04	0.01	0.03	0.00	0.02	0.00	0.04	0.00	0.01	0.01	0.02	0.02
Eubacterium brachy group	0.03	0.00	0.03	0.01	0.04	0.00	0.02	0.01	0.02	0.01	0.02	0.01	0.03	0.04	0.02	0.02
p-1088-a5 gut group	0.00	0.00	0.01	0.00	0.04	0.03	0.04	0.02	0.02	0.01	0.03	0.02	0.01	0.02	0.04	0.02
Campylobacter	0.07	0.05	0.03	0.04	0.02	0.01	0.03	0.01	0.02	0.01	0.01	0.00	0.00	0.01	0.01	0.00
Senegalimassilia	0.00	0.01	0.01	0.01	0.01	0.02	0.01	0.02	0.01	0.02	0.02	0.02	0.04	0.04	0.04	0.03
Prevotella 2	0.02	0.03	0.01	0.03	0.00	0.03	0.00	0.07	0.01	0.03	0.03	0.03	0.01	0.01	0.01	0.01
Elusimicrobium	0.04	0.01	0.00	0.00	0.05	0.01	0.02	0.00	0.03	0.00	0.03	0.01	0.02	0.01	0.03	0.04
Oscillospira	0.00	0.00	0.00	0.01	0.01	0.04	0.01	0.07	0.01	0.05	0.01	0.02	0.00	0.00	0.05	0.02

Enterobacter	0.00	0.00	0.00	0.00	0.00	0.00	0.00	0.01	0.00	0.00	0.00	0.00	0.00	0.00	0.12	0.15
Bacteroidales **	0.01	0.06	0.01	0.01	0.02	0.01	0.01	0.03	0.01	0.00	0.03	0.01	0.02	0.02	0.02	0.02
Lachnospiraceae NK4B4 group	0.00	0.00	0.00	0.00	0.05	0.00	0.02	0.02	0.05	0.02	0.04	0.03	0.03	0.02	0.01	0.00
Ruminococcaceae UCG-001	0.00	0.00	0.00	0.00	0.00	0.00	0.02	0.00	0.04	0.06	0.03	0.01	0.03	0.02	0.03	0.04
Spirochaeta 2	0.00	0.00	0.00	0.00	0.00	0.00	0.01	0.00	0.01	0.00	0.02	0.00	0.01	0.01	0.09	0.12
Lachnospiraceae UCG-009	0.00	0.00	0.00	0.00	0.00	0.00	0.01	0.01	0.02	0.04	0.07	0.04	0.03	0.01	0.01	0.02
Rhizobium	0.00	0.00	0.00	0.00	0.00	0.00	0.01	0.01	0.01	0.01	0.01	0.02	0.01	0.01	0.09	0.08
Asteroleplasma	0.01	0.03	0.02	0.05	0.01	0.01	0.01	0.01	0.02	0.02	0.01	0.03	0.00	0.00	0.01	0.01
Neisseriaceae **	0.05	0.04	0.02	0.03	0.01	0.02	0.02	0.01	0.01	0.01	0.00	0.00	0.01	0.01	0.01	0.01
Lachnobacterium	0.00	0.00	0.00	0.00	0.00	0.00	0.00	0.00	0.00	0.00	0.00	0.00	0.13	0.10	0.01	0.01
Oligosphaeraceae *	0.00	0.00	0.00	0.00	0.00	0.00	0.00	0.00	0.00	0.00	0.04	0.00	0.02	0.03	0.07	0.05
Lachnospiraceae *	0.01	0.01	0.00	0.01	0.01	0.01	0.00	0.01	0.01	0.05	0.03	0.05	0.01	0.01	0.01	0.01
Bacteroidetes BD2-2 *	0.00	0.00	0.00	0.00	0.00	0.00	0.00	0.00	0.00	0.01	0.07	0.01	0.06	0.03	0.01	0.02
Lachnospiraceae NC2004 group	0.00	0.00	0.00	0.00	0.03	0.01	0.01	0.00	0.00	0.00	0.03	0.00	0.05	0.04	0.03	0.03
Lachnoclostridium 1	0.00	0.00	0.04	0.00	0.01	0.01	0.01	0.01	0.01	0.01	0.01	0.00	0.06	0.04	0.01	0.01
Pseudomonas	0.00	0.00	0.00	0.00	0.00	0.01	0.00	0.01	0.01	0.01	0.00	0.01	0.05	0.02	0.06	0.04
hoa5-07d05 gut group	0.00	0.00	0.00	0.02	0.02	0.01	0.04	0.02	0.01	0.02	0.02	0.03	0.00	0.00	0.01	0.01
Family XIII UCG-002	0.00	0.00	0.00	0.00	0.00	0.00	0.01	0.01	0.01	0.01	0.01	0.01	0.02	0.04	0.06	0.05
Veillonellaceae **	0.01	0.01	0.02	0.00	0.05	0.00	0.02	0.00	0.00	0.00	0.00	0.00	0.02	0.02	0.03	0.02
Cloacibacillus	0.01	0.01	0.01	0.01	0.03	0.01	0.02	0.03	0.02	0.01	0.05	0.00	0.00	0.00	0.01	0.01
Chloroplast *	0.00	0.00	0.00	0.00	0.01	0.01	0.01	0.01	0.01	0.01	0.02	0.01	0.06	0.04	0.01	0.01
Christensenellaceae **	0.00	0.00	0.01	0.00	0.01	0.00	0.02	0.00	0.03	0.00	0.03	0.00	0.02	0.02	0.02	0.01
Ruminococcaceae UCG-012	0.00	0.00	0.00	0.00	0.00	0.00	0.01	0.00	0.01	0.00	0.07	0.00	0.01	0.02	0.02	0.03
Methylobacterium	0.00	0.00	0.00	0.00	0.00	0.00	0.00	0.00	0.00	0.01	0.01	0.01	0.01	0.01	0.06	0.07
Sphingomonas	0.00	0.00	0.00	0.00	0.00	0.00	0.00	0.00	0.00	0.02	0.02	0.02	0.03	0.03	0.02	0.02
Lachnospiraceae UCG-010	0.01	0.02	0.00	0.02	0.01	0.01	0.01	0.01	0.01	0.02	0.01	0.02	0.01	0.00	0.01	0.01
Succinivibrionaceae **	0.00	0.00	0.00	0.01	0.01	0.01	0.01	0.01	0.01	0.01	0.02	0.02	0.02	0.01	0.01	0.01

Ruminococcaceae UCG-009	0.00	0.00	0.01	0.01	0.01	0.01	0.02	0.02	0.01	0.01	0.01	0.01	0.01	0.01	0.01	0.01
Enterorhabdus	0.00	0.00	0.00	0.00	0.00	0.00	0.01	0.01	0.00	0.01	0.01	0.01	0.04	0.04	0.01	0.01
Peptococcaceae **	0.00	0.00	0.00	0.00	0.01	0.00	0.02	0.00	0.01	0.00	0.03	0.00	0.01	0.01	0.02	0.02
Oligoflexales *	0.00	0.00	0.00	0.00	0.00	0.00	0.00	0.00	0.00	0.00	0.00	0.00	0.00	0.00	0.10	0.04
Ruminiclostridium 1	0.00	0.00	0.00	0.00	0.00	0.00	0.00	0.00	0.00	0.00	0.01	0.00	0.03	0.02	0.04	0.04
Pseudoramibacter	0.01	0.00	0.02	0.02	0.03	0.02	0.02	0.02	0.01	0.00	0.00	0.00	0.00	0.00	0.00	0.00
Corynebacterium 1	0.01	0.01	0.00	0.01	0.00	0.01	0.01	0.02	0.01	0.02	0.01	0.01	0.01	0.01	0.00	0.00
Rhodococcus	0.00	0.00	0.00	0.00	0.01	0.01	0.00	0.00	0.00	0.00	0.00	0.01	0.02	0.01	0.04	0.04
Candidatus Hepatincola	0.00	0.01	0.00	0.00	0.00	0.00	0.00	0.00	0.00	0.01	0.01	0.02	0.00	0.00	0.05	0.04
Oscillibacter	0.00	0.00	0.00	0.02	0.00	0.01	0.01	0.04	0.00	0.02	0.00	0.01	0.00	0.00	0.00	0.00
Allisonella	0.03	0.03	0.02	0.02	0.00	0.00	0.00	0.00	0.00	0.00	0.00	0.00	0.01	0.01	0.00	0.00
Lachnospiraceae UCG-004	0.01	0.00	0.02	0.01	0.01	0.01	0.00	0.00	0.00	0.00	0.01	0.00	0.02	0.02	0.01	0.01
Erysipelothrix	0.00	0.00	0.00	0.00	0.01	0.01	0.01	0.01	0.03	0.02	0.01	0.01	0.00	0.00	0.00	0.00
Lachnoclostridium 10	0.00	0.00	0.00	0.00	0.00	0.00	0.00	0.00	0.00	0.00	0.00	0.01	0.00	0.01	0.04	0.04
Lachnoclostridium 12	0.00	0.00	0.00	0.00	0.00	0.00	0.00	0.00	0.00	0.00	0.00	0.00	0.06	0.01	0.01	0.02
Acinetobacter	0.01	0.00	0.00	0.01	0.01	0.01	0.01	0.00	0.01	0.01	0.00	0.01	0.01	0.01	0.01	0.01
Tyzzerella	0.00	0.01	0.00	0.01	0.00	0.01	0.01	0.01	0.00	0.01	0.00	0.02	0.00	0.00	0.00	0.00
Anaerobiospirillum	0.00	0.01	0.02	0.01	0.00	0.01	0.00	0.00	0.00	0.00	0.00	0.00	0.00	0.00	0.01	0.01
Ruminococcaceae UCG-008	0.00	0.00	0.00	0.01	0.01	0.02	0.00	0.02	0.00	0.02	0.01	0.00	0.00	0.00	0.00	0.00
Acholeplasma	0.03	0.00	0.00	0.00	0.00	0.03	0.00	0.01	0.00	0.00	0.00	0.00	0.00	0.01	0.00	0.00
Erysipelotrichaceae UCG-008	0.00	0.00	0.00	0.00	0.00	0.00	0.00	0.00	0.01	0.00	0.01	0.00	0.01	0.01	0.02	0.02
PL-11B10 *	0.00	0.00	0.00	0.00	0.00	0.01	0.00	0.02	0.01	0.01	0.00	0.01	0.00	0.00	0.01	0.02
Collinsella	0.00	0.00	0.00	0.00	0.00	0.01	0.00	0.01	0.00	0.02	0.00	0.01	0.00	0.01	0.00	0.00
Mannheimia	0.00	0.01	0.00	0.01	0.00	0.01	0.00	0.00	0.01	0.01	0.00	0.00	0.01	0.00	0.01	0.00
Denitrobacterium	0.00	0.00	0.00	0.02	0.00	0.01	0.00	0.01	0.00	0.00	0.00	0.01	0.01	0.02	0.00	0.00
Faecalibacterium	0.05	0.00	0.00	0.00	0.00	0.00	0.00	0.00	0.00	0.00	0.00	0.00	0.00	0.00	0.00	0.00
Eubacterium	0.02	0.03	0.01	0.01	0.00	0.01	0.00	0.00	0.00	0.01	0.00	0.00	0.00	0.00	0.00	0.00

Erwinia	0.00	0.00	0.00	0.00	0.00	0.00	0.00	0.00	0.00	0.00	0.00	0.00	0.01	0.00	0.03	0.03
Parvibacter	0.00	0.00	0.00	0.00	0.00	0.00	0.00	0.00	0.00	0.00	0.00	0.00	0.02	0.03	0.01	0.01
Other F_PeH15	0.00	0.00	0.00	0.00	0.00	0.00	0.00	0.00	0.00	0.00	0.00	0.00	0.00	0.00	0.03	0.04
Comamonas	0.01	0.02	0.00	0.00	0.01	0.01	0.00	0.00	0.00	0.00	0.00	0.00	0.00	0.00	0.00	0.00
Odoribacter	0.01	0.01	0.00	0.01	0.00	0.00	0.00	0.00	0.00	0.00	0.02	0.01	0.00	0.00	0.00	0.00
Curtobacterium	0.00	0.00	0.00	0.00	0.00	0.00	0.00	0.00	0.00	0.01	0.01	0.01	0.00	0.00	0.01	0.01
Porphyromonas	0.01	0.03	0.01	0.01	0.00	0.00	0.00	0.00	0.00	0.00	0.00	0.00	0.00	0.00	0.00	0.00
possible genus Sk018	0.00	0.00	0.00	0.00	0.00	0.00	0.00	0.00	0.00	0.00	0.00	0.00	0.03	0.01	0.01	0.01
Armatimonadetes *	0.00	0.00	0.00	0.00	0.00	0.00	0.00	0.00	0.00	0.00	0.01	0.00	0.01	0.01	0.02	0.02
Fretibacterium	0.00	0.00	0.00	0.00	0.00	0.00	0.02	0.00	0.01	0.00	0.00	0.00	0.01	0.00	0.01	0.01
UCT N117 *	0.00	0.00	0.00	0.00	0.01	0.00	0.01	0.00	0.01	0.00	0.01	0.00	0.00	0.00	0.01	0.02
Intestinimonas	0.00	0.00	0.00	0.00	0.00	0.00	0.00	0.00	0.00	0.00	0.00	0.00	0.04	0.01	0.00	0.00
Haemophilus	0.00	0.00	0.00	0.00	0.00	0.01	0.00	0.01	0.01	0.01	0.00	0.01	0.00	0.00	0.00	0.00
Coprococcus 3	0.00	0.00	0.00	0.00	0.00	0.01	0.00	0.01	0.00	0.00	0.02	0.00	0.00	0.00	0.00	0.00
Flavobacterium	0.00	0.00	0.00	0.00	0.00	0.01	0.00	0.00	0.01	0.01	0.00	0.00	0.01	0.01	0.00	0.00
Candidatus Soleaferrea	0.01	0.00	0.00	0.01	0.00	0.00	0.00	0.00	0.00	0.00	0.01	0.00	0.00	0.00	0.00	0.00
Microbacterium	0.00	0.00	0.00	0.00	0.00	0.00	0.00	0.00	0.00	0.00	0.01	0.01	0.00	0.01	0.01	0.01
Tyzzerella 4	0.01	0.00	0.01	0.00	0.01	0.00	0.00	0.01	0.00	0.00	0.00	0.01	0.00	0.00	0.00	0.00
Bibersteinia	0.00	0.00	0.00	0.00	0.00	0.00	0.00	0.00	0.00	0.00	0.00	0.00	0.01	0.01	0.02	0.01
Christensenella	0.01	0.01	0.01	0.01	0.00	0.01	0.00	0.00	0.00	0.00	0.00	0.00	0.00	0.00	0.00	0.00
Brevundimonas	0.00	0.00	0.00	0.00	0.01	0.01	0.00	0.00	0.01	0.01	0.00	0.01	0.00	0.00	0.00	0.00
MSBL5 *	0.00	0.00	0.00	0.00	0.00	0.00	0.00	0.00	0.00	0.00	0.00	0.00	0.02	0.00	0.02	0.01
Neisseria	0.01	0.01	0.00	0.00	0.00	0.01	0.00	0.00	0.00	0.01	0.00	0.00	0.00	0.00	0.00	0.00
WCHB1-25 *	0.00	0.00	0.00	0.01	0.00	0.00	0.00	0.00	0.00	0.00	0.00	0.00	0.00	0.00	0.01	0.00
Desulfobulbus	0.00	0.00	0.00	0.00	0.00	0.01	0.00	0.00	0.00	0.01	0.00	0.00	0.00	0.00	0.00	0.00
Marinicella	0.00	0.00	0.00	0.00	0.00	0.00	0.00	0.01	0.01	0.01	0.01	0.01	0.00	0.00	0.00	0.00
Fusicatenibacter	0.00	0.00	0.00	0.01	0.00	0.00	0.00	0.00	0.00	0.00	0.01	0.00	0.00	0.00	0.00	0.00

Bacillus	0.00	0.00	0.00	0.00	0.00	0.00	0.00	0.00	0.00	0.00	0.00	0.00	0.00	0.00	0.01	0.02
Rahnella	0.00	0.00	0.00	0.00	0.00	0.00	0.00	0.00	0.00	0.00	0.00	0.00	0.00	0.00	0.02	0.02
Catonella	0.00	0.00	0.00	0.00	0.00	0.00	0.00	0.00	0.00	0.00	0.00	0.00	0.02	0.02	0.00	0.00
Hafnia	0.00	0.00	0.00	0.00	0.00	0.00	0.00	0.00	0.00	0.00	0.00	0.00	0.00	0.00	0.02	0.03
Tatumella	0.00	0.00	0.00	0.00	0.00	0.00	0.00	0.00	0.00	0.00	0.00	0.00	0.00	0.00	0.02	0.02
WA-aaa01f12 *	0.00	0.00	0.00	0.00	0.00	0.00	0.00	0.00	0.00	0.00	0.02	0.00	0.00	0.00	0.01	0.01
na	0.14	0.17	0.13	0.14	0.17	0.22	0.19	0.21	0.18	0.20	0.30	0.22	0.35	0.32	0.44	0.41

* Unclassified genera

** Uncultured genera
